# Insights into cranial anatomy and craniometry of the aardwolf (*Proteles cristata*) with comparisons to extant hyaenids

**DOI:** 10.1186/s12917-026-05290-5

**Published:** 2026-01-26

**Authors:** Krzysztof Stegmann, Shaw Badenhorst, Wojciech Borawski, Dominik Poradowski, Maciej Janeczek, Anna Mucha, Joanna Klećkowska-Nawrot

**Affiliations:** 1 RZGW, C. K. Norwida 34, Wrocław, 50-950 Poland; 2 Evolutionary Studies Institute, University of the Witwatersrand, 1 Jan Smuts Ave, Braamfontein, Johannesburg, 2000 South Africa; 3 Department and Surgery Clinic, Faculty of Veterinary Medicine, Wroclaw University of Environmental and Life Sciences, Grunwaldzki Sq. 51, Wrocław, 50-366 Poland; 4 Department of Biostructure and Animal Physiology, Faculty of Veterinary Medicine, Wrocław University of Environmental and Life Sciences, Kożuchowska 1, Wrocław, 51-631 Poland; 5 Department of Genetics, Wrocław University of Environmental and Life Sciences, Kożuchowska 7, Wrocław, 51-631 Poland

**Keywords:** Brown hyena, Mandible, Morphology, Morphometry, Skull, Spotted hyena, Striped hyena

## Abstract

**Background:**

The aardwolf skulls were analyzed and compared to the spotted hyena, the brown hyena and the striped hyena, using specimens from Polish and South African collections. Addressing gaps in detailed anatomy and morphometrics, this study used extensive analyzes, encompassing 64 morphometric parameters and 7 indices, to examine the morphology and morphometrics of cranial and mandibular structures and quantify interspecific and intraspecific variation.

**Results:**

The comparative analysis of hyena skulls revealed significant differences between the aardwolf and the other three species. The aardwolf consistently had the smallest and proportionally narrowest skull, characterized by high morphological stability and a wide neurocranium. The brown hyena and spotted hyena had the largest skulls; however, the brown hyena showed greater homogeneity, while the spotted hyena displayed higher absolute variability. Morphologically, the aardwolf’s basilar part was cylindrical, unlike the pyramidal shape in the spotted hyena, and it possessed prominent nuchal tubercles that were reduced in the brown and spotted hyenas.

**Conclusions:**

This study demonstrates that the morphological and morphometric features of the aardwolf’s skull are highly specialized and fundamentally distinct from the other three hyena species, reflecting its unique dietary niche. The powerful skulls of the spotted and brown hyenas, in contrast, correlate with their roles as apex predators and scavengers.

## Background

The carnivore family Hyaenidae includes four extant representatives: the smallest one – aardwolf (*Proteles cristata*, Sparrman, 1783), also called the maanhaar-jackal, termite-eating hyena, or civet hyena; found in Africa and in southwestern and south-central Asia; the spotted hyena (*Crocuta crocuta*, Erxleben, 1777), also known as the laughing hyena; the brown hyena (*Parahyaena brunnea*, Thunberg, 1820), also called strandwolf; the striped hyena (*Hyaena hyaena*, Linnaeus, 1758) [1].

The four extant hyena species included in this study exhibit varying conservation statuses. According to IUCN Red List (2024-2), the Aardwolf is globally classified as Least Concern (LC) with a stable population [2], and its Mediterranean population also has a Least Concern (LC) status [3]. The spotted hyena is currently classified as Least Concern (LC), though its population is experiencing a decline [4]. The brown hyena is categorized as Near Threatened (NT) with a stable population [5]. The striped hyena has a global status of Near Threatened (NT) with a decreasing population [6], while its Mediterranean population is considered Vulnerable (VU) [7].

Hyaenidae are often perceived merely as scavengers; however, their diet is remarkably diverse, including not only carrion, but also fresh kills, plant matter, and more. All four extant species rely on an animal-based diet (whether strictly insectivorous or predominantly carnivorous), the composition of which varies significantly depending on the species [8–12]. Aardwolves generally feed on termites and larvae (mainly *Trinervitermes* and also *Hodotermes*), and less on other insects [8, 9, 11, 12]. They are often mistakenly accused of killing livestock such as goats and sheep, a behavior unlikely given their reduced dentition [12]. They are reported to consume carrion [11], but no good evidence has been found [9]. Spotted hyenas primarily consume medium-sized and large ungulates, such as wildebeest, zebra, antelope, buffalo, gemsbok, springbok, steenbok, eland, kudu and impala. Their diet includes mammals ranging down to the size of mice. Forty-three different food items were recorded [8, 9, 12]. The diet of brown hyenas is diverse and opportunistic, consisting mainly of carrion from major predators, supplemented by small vertebrates, birds’ eggs, various invertebrates, and a variety of plant matter, including fruits, vegetables, and fungi [8–12]. Along the Namibian coast, fish and seals are also part of their diet, thus they are often called a strandwolf or a strandloper [9]. Up to 58 different food items have been documented [10]. Another scavenger, the striped hyena, exhibits an omnivorous diet that includes both animal matter (insects and small vertebrates) and plant matter (figs, desert dates), as well as carrion [9, 12]. Sporadically, they may kill small domestic stock [9], though they rarely prey on animals larger than newborn antelopes [10].

Hyenas have adapted to various environments through numerous morphological adaptations. For instance, spotted hyenas possess remarkably powerful jaws, which enable them to crush even large bones, facilitating the extraction of marrow and the swallowing of substantial pieces of meat and bone [9] (a trait for which they are well known, even among nonspecialists). In addition to eating bones, they can also eat and digest hides, horns, and teeth. Their powerful build, characterized by strong shoulders that are higher than their hindquarters, assists them in digging and chasing prey [10]. Similarly to other carnivores, hyenas play a crucial role in regulating animal populations (demonstrating more complete and efficient resource utilization than any other carnivore), removing carcasses, thus preventing the spread of disease and dispersing seeds through fruit consumption [10].

Numerous morphological and morphometrical studies have further illuminated the diverse structural characteristics of hyenas (Table 1). These investigations have examined not only the overall size of the body and individual bones but also their intricate shapes and detailed architecture. For example, while the robust build and powerful jaws of the spotted hyena are well-documented adaptations for hunting larger prey (as previously mentioned), the aardwolf, in stark contrast, exhibits a considerably smaller body size, a reduced number of diminutive teeth, and weak jaws specifically adapted for its insectivorous diet. However, while there are numerous publications, a comprehensive understanding of the classical anatomy could be further enhanced by more detailed descriptions, and many morphometric parameters present opportunities for comparative investigation.

**Table 1:** Overview of the literature that includes anatomical and morphometric analysis of the skull for spotted hyena, brown hyena, striped hyena and aardwolf. If skull measurements were performed in these studies, they are listed along with the number of measurements taken

Species				Measurements	No of measurements	Reference
Spotted hyena	Brown hyena	Striped hyena	Aardwolf			
		x		skull base, skull width, palatal length, fascial length, cranial length, cranial width, total length, total height, candular height, length of the dental series	10	[13]
x						[14]
x				head circumference, head width, head length, ear to canine, ear to third premolar, tooth row, canine height, tooth lengths	8	[15]
x	x	x		gape: clearance between upper and lower canine at maximum gape, gape dimension; jaw joint to: canine, carnassial; lower canine: anteroposterior diameter, length, lateromedial diameter; lower jaw: length, moment arm of masseter muscle, moment arm of temporalis muscle; skull: basicranial length, length, snout length; upper canine: anteroposterior diameter, length, lateromedial diameter	16	[16]
x	x	x				[17]
x	x	x				[18]
		x	x			[19]
x			x			[20]
x			x			[21]
x	x	x	x	condylobasal length, facial flexion	2	[22]
x	x	x				[23]
		x				[24]
		x				[25]
x			x	skull: condylo-basal length, length of nasals, length of the muzzle, height of muzzle, length of palate, breadth of palate, maximum breath of palate, length of pterygoid, aperture of pterygoid, length of upper postcanine tooth row, maximum breadth zygomatic, breadth of braincase, breadth of bulla, length of bulla, length of orbit, height of occipital plate, postorbital constriction, mastoid width. Mandible: length of dentary, height of dentary, length of coronoid process, height of coronoid process.	22	[26]
						[27]
x						[28]
x						[29]
x						[30]
x						[31]
x	x			zygomatic arch width, occipital width, temporal fossa width, temporal fossa length, tooth row length, jaw length, condyle to M1 length, moment arm of temporalis, moment arm of masseter, masseteric fossa length, occipital height	11	[32]
x	x	x	x			[33]
x	x		x			[34]

In this paper, we analyze the cranial and mandibular anatomy of the aardwolf, along with selected morphometric parameters, and compare it to the other three extant hyenas. To objectively quantify the morphological distinctiveness among the four species, we employ Principal Component Analysis (PCA) on the craniometric data. A thorough understanding of the anatomy of the cranial and mandibular is essential knowledge for veterinarians, zookeepers and other specialists working with these animals in zoological institutions. Our results provide valuable information that can assist in diagnosing pathological conditions, performing surgical procedures and ensuring their general health in daily care.

## Materials and methods

### Specimen collection

**Fig. 1:**
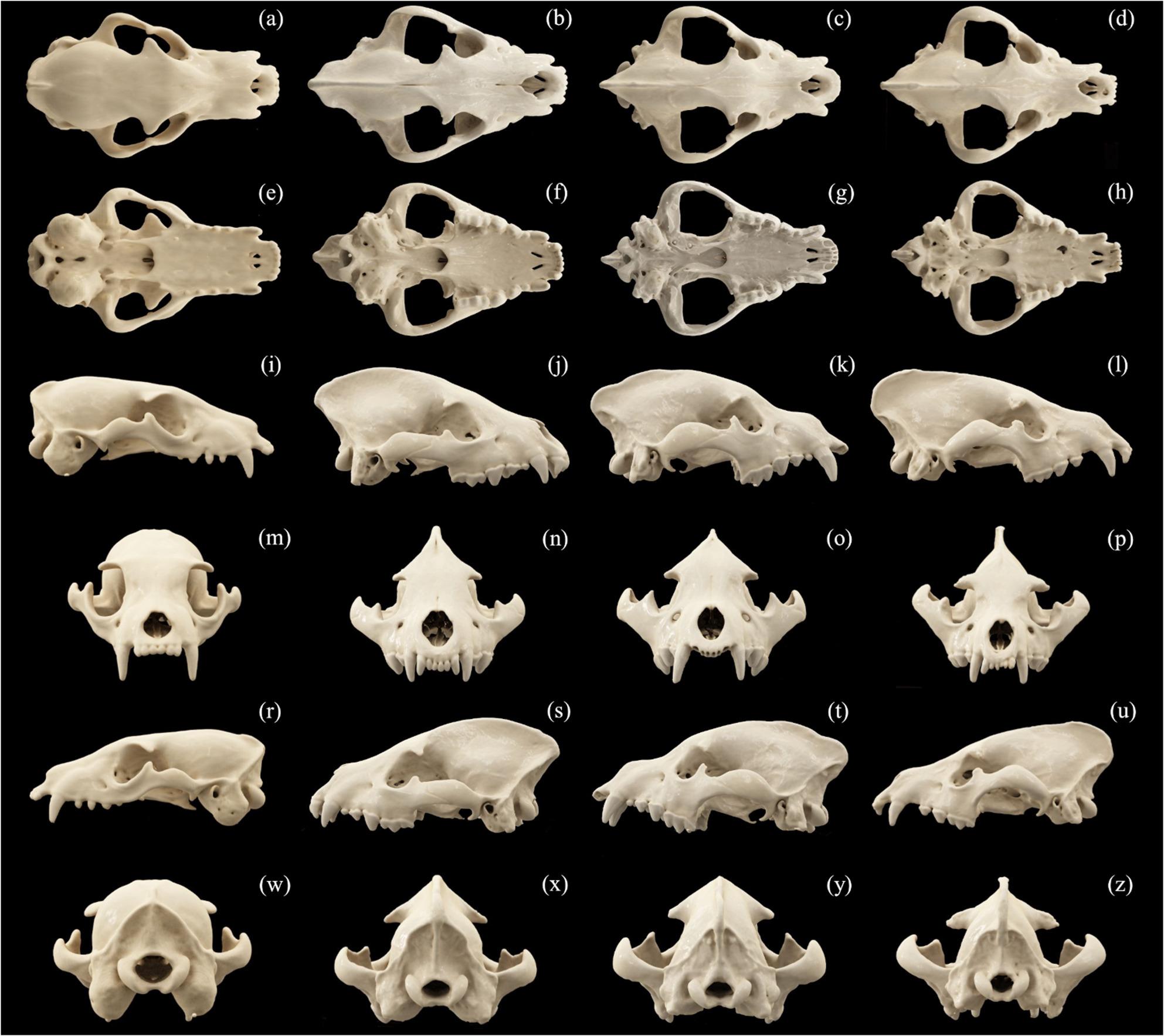
Comparative arrangement of skull CT scans of the aardwolf (**a**, **e**, **i**, **m**, **r**, **w**), the spotted hyena (**b**, **f**, **j**, **n**, **s**, **x**), the brown hyena (**c**, **g**, **k**, **o**, **t**, **y**) and the striped hyena (**d**, **h**, **l**, **p**, **u**, **z**) in different views: dorsal view (**a**, **b**, **c**, **d**); basal view (**e**, **f**, **g**, **h**); right side view (**i**, **j**, **k**, **l**); nuchal view (**m**, **n**, **o**, **p**); left side view (**r**, **s**, **t**, **u**) and nasal view (**w**, **x**, **y**, **z**)

This study is based on two collections: those housed at the Department of Biostructure and Animal Physiology of the Faculty of Veterinary Medicine at the Wrocław University of Environmental and Life Sciences, Poland (WUELS), and of the Evolutionary Studies Institute at the University of the Witwatersrand, Johannesburg, South Africa (ESIUW). A total of 12 adult skulls (Fig. 1) were analyzed: aardwolves (*n* = 3), spotted hyenas (*n* = 5), striped hyena (*n* = 1) and brown hyenas (*n* = 3) were analyzed. The specimens studied are: *Proteles cristata*: ESIUW BPI/C/240, BPI/C/241; WUELS: H/PC/1/C; *Crocuta crocuta*: ESIUW BPI/C/202, BPI/C/203; WUELS H/CC/1/C, H/CC/57/C, H/CC/513/C; *Hyaena hyaena*: WUELS H/HH/1/C; *Parahyaena brunnea*: ESIUW BPI/C/204, BPI/4/1157; WUELS: H/PB/1/C.

### Morphological procedures

Morphological analysis follows *Nomina Anatomica Veterinaria* [35] and Lehrbuch der Anatomie der Haustiere [36], focusing on bones visible externally without altering the integrity of the skulls. Consequently, certain internal structures, such as the ethmoid bone, ventral nasal concha bone, and rostral bone, were not described. Furthermore, hyoid apparatus was not present in the analyzed samples and therefore was not included in the study. Digital images were acquired using two cameras in a lightbox setup: a Nikon D300s with a Tamron SP AF 17–50 mm f/2.8 XR Di II LD Aspherical (IF) lens, a Lumix DMC-GX80 camera with a Panasonic G VARIO 12–32 mm f/3.5–5.6 ASPH. M.O.I.S. lens, and a Siemens go.TOP 64-slice, 128-layer CT scanner. Scanning of 4 skulls (one of each species from the WUELS collection) was conducted along the skull’s long axis, first in the nasal direction and then in the caudal direction. Each skull was individually scanned. The scan parameters were as follows: 120 kV tube voltage of 120 kV and tube current of 60 mAs. Image processing and reconstruction were carried out using Siemens’ syngo.via software. Multiplanar reconstructions (MPR) were performed in sagittal, dorsal, and axial planes were performed, as well as three-dimensional (3D) volume rendering for comprehensive visualization. The images were subsequently processed using GIMP version 2.10.38.

### Morphometric procedures

**Fig. 2:**
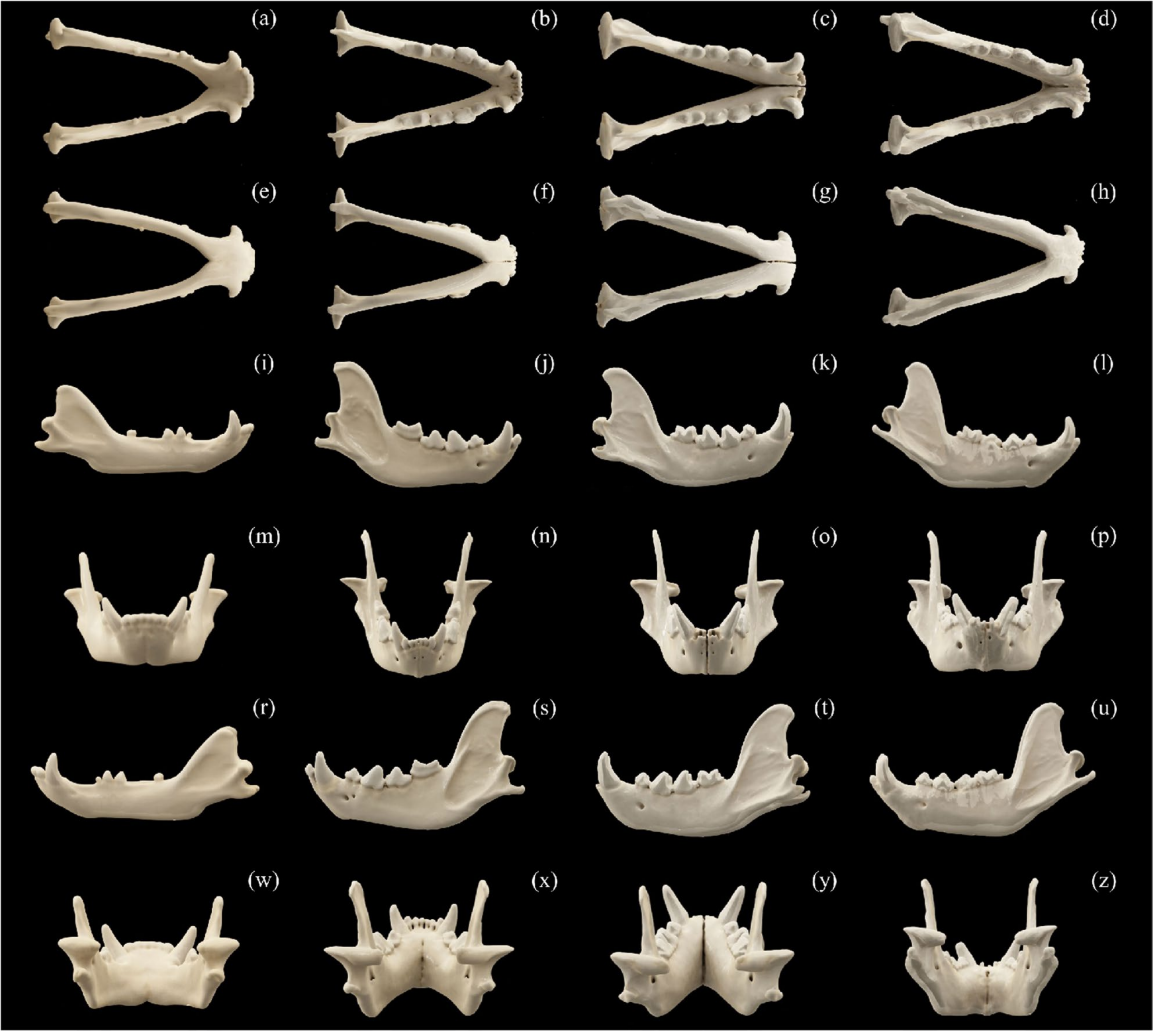
Comparative arrangement of mandible CT scans of the aardwolf (**a**, **e**, **i**, **m**, **r**, **w**), the spotted hyena (**b**, **f**, **j**, **n**, **s**, **x**), the brown hyena (**c**, **g**, **k**, **o**, **t**, **y**) and the striped hyena (**d**, **h**, **l**, **p**, **u**, **z**) in different views: dorsal view (**a**, **b**, **c**, **d**); basal view (**e**, **f**, **g**, **h**); right side view (**i**, **j**, **k**, **l**); nuchal view (**m**, **n**, **o**, **p**); left side view (**r**, **s**, **t**, **u**) and nasal view (**w**, **x**, **y**, **z**)

**Fig. 3:**
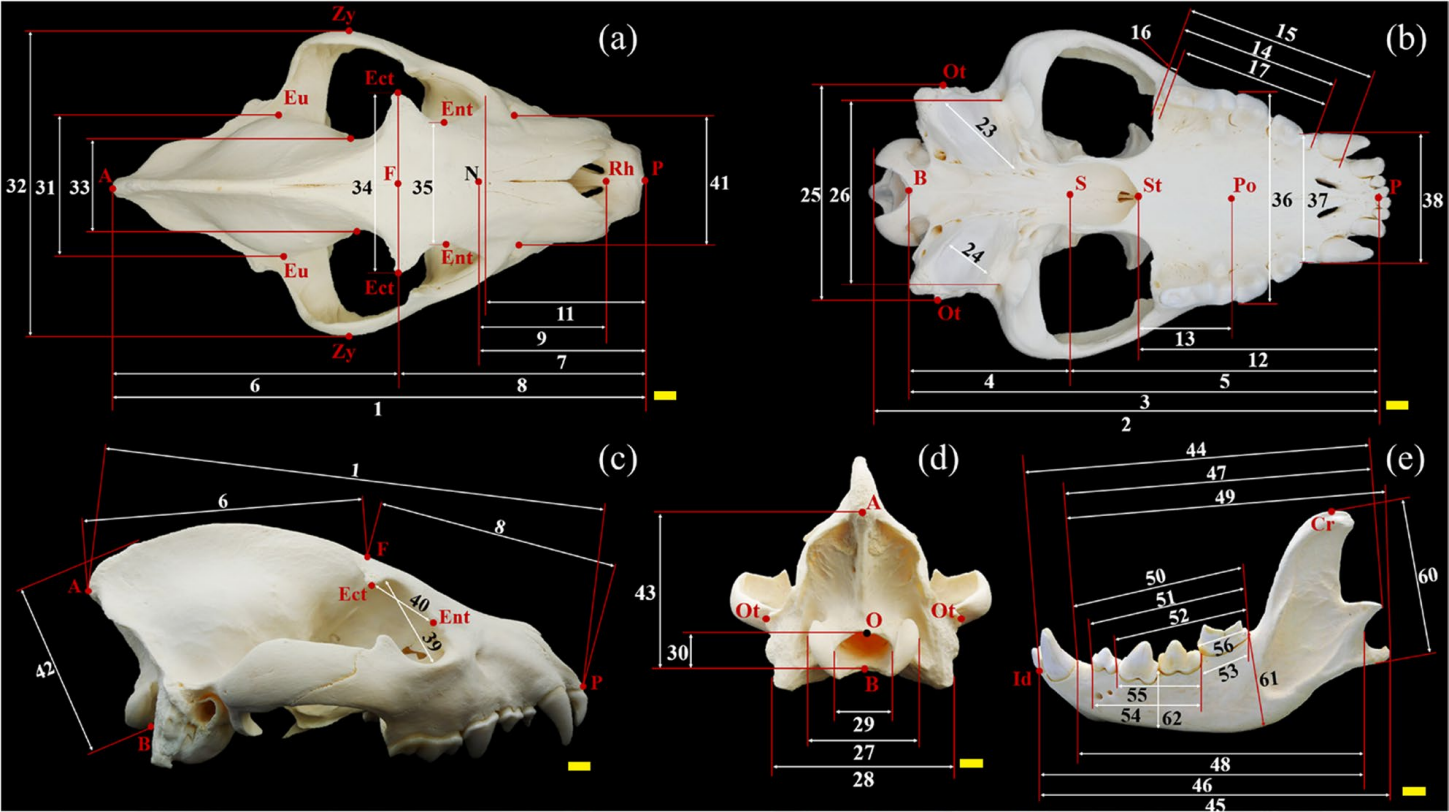
Skull measurements on the example of the spotted hyena. Dorsal view (**a**); basal view (**b**); right side view (**c**); nuchal view (**d**); left side view (**e**). Scale bar (yellow) = 1 cm. A – Akrokranion (a,c,d), B – Basion (b,c,d), Cr – Coronion (e), Ect – Ectorbitale (a,c), Ent – Entorbitale (a,c), Eu – Euryon (a), F – Frontal midpoint (a,c), Id – Infradentale (e), N – Nasion (a), O – Opisthion (d), Ot – Otion (b,d), P – Prosthion (a,b,c), Po – Palatinoorale (b), Rh – Rhinion (a), S – Synsphenion (b), St – Staphylion (b), Zy – Zygion (a)

Morphometric analysis follows von den Driesch [37] and Duerst [38]. Different skull parameters (Table 2) were obtained using digital calipers: two 150 mm calipers (0.1 mm resolution, ± 0.2 mm accuracy), one 300 mm caliper (0.1 mm resolution, ± 0.3 mm accuracy), and a Martin breadth caliper (accuracy ± 5 mm). All measurements were taken in millimeters (mm). For each specimen, up to six measurements were taken per parameter; the average of these six measurements was used as the final metric for that individual in order to minimize measurement error. A total of 64 measurements were taken (Figs. 2 and 3). Additionally, seven indices were calculated (Table 3), following von den Driesch [37] and Onar et al. [39], using Microsoft^®^ Excel^®^ LTSC MSO (version 2408, build 16.0.17932.20328) 64-bit. These indices quantify key cranial proportions and provide valuable comparative data that may assist veterinary and zoological specialists in assessing growth anomalies or pathological changes in individual animals.

**Table 2:** Morphometric parameters taken, following von Den Driesch [37]

Region	Parameter	Description
Skull measurements	**1**	Total length: Akrokranion – Prosthion
	**2**	Condylobasal length: aboral border of the occipital condyles – Prosthion
	**3**	Basal length: Basion – Prosthion
	**25**	Greatest mastoid = greatest breadth of the occipital triangle: Otion – Otion
	**26**	Breadth dorsal to the external auditory meatus
	**27**	Greatest breadth of the occipital condyles
	**28**	Greatest breadth of the bases of the paraoccipital processes
	**32**	Zygomatic breadth: Zygion – Zygion
Cranial measurements	**4**	Basicranial axis: Basion – Synsphenion
	**6**	Upper neurocranium length: Akrokranion – Frontal midpoint
	**10**	Length of the braincase. This measurement can be taken only when the cribriform plate is preserved. Insert a thin ruler through the foramen magnum. The front end must reach the cribriform plate and the measurement is read off against the Basion. (Not shown in Fig. 3)
	**31**	Greatest neurocranium width = greatest breadth of braincase: Euryon – Euryon
	**33**	Least breadth of skull = least breadth aboral of the supraorbital processes = breadth at the postorbital constriction
	**42**	Skull height: measured from the base of the skull (on the basioccipital) to the highest elevation of the sagittal crest
	**43**	Height of the occipital triangle: Akrokranion – Basion
Foramen magnum measurements	**29**	Greatest breadth of the foramen magnum
	**30**	Height of the foramen magnum: Basion – Opisthion
Orbital measurements	**34**	Frontal breadth: Ectorbitale – Ectorbitale
	**35**	Least breadth between the orbits: Entorbitale – Entorbitale
	**39**	Greatest inner height of the orbit
	**40**	Greatest inner length of the orbit: Ectorbitale – Entorbitale
Tympanic bulla measurements	**23**	Greatest diameter of the auditory bulla: from the most aboral point of the bulla on the suture with the paraoccipital process up to the external carotid foramen
	**24**	Least diameter of the auditory bulla
Facial measurements	**5**	Basifacial axis: Synsphenion – Prosthion
	**7**	Viscerocranium length: Nasion – Prosthion
	**8**	Facial length: Frontal midpoint – Prosthion
	**9**	Greatest length of the nasals: Nasion – Rhinion
	**11**	“Snout” length: oral border of the orbits (median) – Prosthion
	**41**	Facial breadth between the infraorbital foramina (least distance)
Palatal measurements	**12**	Median palatal length: Staphylion – Prosthion
	**13**	Length of the horizontal part of the palatine: Staphylion – Palatinoorale
	**14**	Length of the cheektooth row: measured along the alveoli on the buccal side
	**15**	Length of the cheektooth row + canine: measured along the alveoli on the buccal side; aboral border of the alveolus of the first molar - oral border of the alveolus of the canine
	**16**	Length of the molar row: measured along the alveoli on the buccal side
	**17**	Length of the premolar row: measured along the alveoli on the buccal side
	**18**	Length of the carnassial: measured on the buccal side at the cingulum (Not shown in Fig. 3)
	**19**	Greatest breadth of the carnassial (Not shown in Fig. 3)
	**20**	Length of the carnassial alveolus (Not shown in Fig. 3)
	**21**	Length of the first molar: measured on the buccal side at the cingulum (Not shown in Fig. 3)
	**22**	Breadth of the first molar: measured on the buccal side at the cingulum (Not shown in Fig. 3)
	**36**	Greatest palatal breadth: measured across the outer borders of the alveoli
	**37**	Least palatal breadth: measured behind the canines
	**38**	Breadth at the canine alveoli
	**12a**	Palatal length: the median point of intersection of the line joining the deepest indentations of the Choanae – Prosthion
	**13a**	Length of the horizontal part of the palatine corresponding to 12a: the median point of intersection of the line joining the deepest indentations of the Choanae – Palatinoorale
Mandible measurements	**44**	Total length: length from the condyle process - Infradentale
	**45**	Length: the angular process - Infradentale
	**46**	Length from the indentation between the condyle process and the angular process - Infradentale
	**47**	Length: the condyle process - aboral border of the canine alveolus
	**48**	Length from the indentation between the condyle process and the angular process - aboral border of the canine alveolus
	**49**	Length: the angular process - aboral border of the canine alveolus
	**50**	Length: the aboral border of the alveolus of the first molar - aboral border of the canine alveolus
	**51**	Length of the cheektooth row, first molar-first premolar: measured along the alveoli
	**52**	Length of the cheektooth row, first molar-second premolar: measured along the alveoli
	**53**	Length of the molar row: measured along the alveoli
	**54**	Length of the premolar row, first premolar-third premolar: measured along the alveoli
	**55**	Length of the premolar row, second premolar-third premolar: measured along the alveoli
	**56**	Length of the carnassial: measured along the cingulum
	**57**	Breadth of the carnassial: measured along the cingulum (Not shown in Fig. 3)
	**58**	Length of the carnassial alveolus (Not shown in Fig. 3)
	**59**	Greatest thickness of the body (below the first molar) (Not shown in Fig. 3)
	**60**	Height of the vertical ramus: basal point of the angular process - Coronion
	**61**	Height of the mandible behind the first molar: measured on the lingual side and at right angles to the basal border
	**62**	Height of the mandible between the second premolar and the third premolar: measured on the lingual side and at right angles to the basal border

**Table 3:** Calculated indexes for the skulls

Index		Formula
I1	Skull index	$\:\frac{\mathrm{z}\mathrm{y}\mathrm{g}\mathrm{o}\mathrm{m}\mathrm{a}\mathrm{t}\mathrm{i}\mathrm{c}\:\mathrm{b}\mathrm{r}\mathrm{e}\mathrm{a}\mathrm{d}\mathrm{t}\mathrm{h}\:\left(\mathrm{P}32\right)}{\mathrm{t}\mathrm{o}\mathrm{t}\mathrm{a}\mathrm{l}\:\mathrm{l}\mathrm{e}\mathrm{n}\mathrm{g}\mathrm{t}\mathrm{h}\:\left(\mathrm{P}1\right)}\:\times\:100$
I2	Facial index 1	$\:\frac{\mathrm{z}\mathrm{y}\mathrm{g}\mathrm{o}\mathrm{m}\mathrm{a}\mathrm{t}\mathrm{i}\mathrm{c}\:\mathrm{b}\mathrm{r}\mathrm{e}\mathrm{a}\mathrm{d}\mathrm{t}\mathrm{h}\:\left(\mathrm{P}32\right)}{\mathrm{v}\mathrm{i}\mathrm{s}\mathrm{c}\mathrm{e}\mathrm{r}\mathrm{o}\mathrm{c}\mathrm{r}\mathrm{a}\mathrm{n}\mathrm{i}\mathrm{u}\mathrm{m}\:\mathrm{l}\mathrm{e}\mathrm{n}\mathrm{g}\mathrm{t}\mathrm{h}\:\left(\mathrm{P}7\right)\:}\:\times\:100$
I3	Facial index 2	$\:\frac{\mathrm{g}\mathrm{r}\mathrm{e}\mathrm{a}\mathrm{t}\mathrm{e}\mathrm{s}\mathrm{t}\:\mathrm{p}\mathrm{a}\mathrm{l}\mathrm{a}\mathrm{t}\mathrm{a}\mathrm{l}\:\mathrm{b}\mathrm{r}\mathrm{e}\mathrm{a}\mathrm{d}\mathrm{t}\mathrm{h}\:\left(\mathrm{P}36\right)}{\mathrm{g}\mathrm{r}\mathrm{e}\mathrm{a}\mathrm{t}\mathrm{e}\mathrm{s}\mathrm{t}\:\mathrm{l}\mathrm{e}\mathrm{n}\mathrm{g}\mathrm{t}\mathrm{h}\:\mathrm{o}\mathrm{f}\:\mathrm{t}\mathrm{h}\mathrm{e}\:\mathrm{n}\mathrm{a}\mathrm{s}\mathrm{a}\mathrm{l}\mathrm{s}\:\left(\mathrm{P}9\right)}\:\times\:100$
I4	Index 1	$\:\frac{\mathrm{g}\mathrm{r}\mathrm{e}\mathrm{a}\mathrm{t}\mathrm{e}\mathrm{s}\mathrm{t}\:\mathrm{n}\mathrm{e}\mathrm{u}\mathrm{r}\mathrm{o}\mathrm{c}\mathrm{r}\mathrm{a}\mathrm{n}\mathrm{i}\mathrm{u}\mathrm{m}\:\mathrm{b}\mathrm{r}\mathrm{e}\mathrm{a}\mathrm{d}\mathrm{t}\mathrm{h}\:\left(\mathrm{P}31\right)}{\mathrm{t}\mathrm{o}\mathrm{t}\mathrm{a}\mathrm{l}\:\mathrm{l}\mathrm{e}\mathrm{n}\mathrm{g}\mathrm{t}\mathrm{h}\:\left(\mathrm{P}1\right)}\:\times\:100$
I5	Index 2	$\:\frac{\mathrm{g}\mathrm{r}\mathrm{e}\mathrm{a}\mathrm{t}\mathrm{e}\mathrm{s}\mathrm{t}\:\mathrm{n}\mathrm{e}\mathrm{u}\mathrm{r}\mathrm{o}\mathrm{c}\mathrm{r}\mathrm{a}\mathrm{n}\mathrm{i}\mathrm{u}\mathrm{m}\:\mathrm{b}\mathrm{r}\mathrm{e}\mathrm{a}\mathrm{d}\mathrm{t}\mathrm{h}\:\left(\mathrm{P}31\right)}{\mathrm{b}\mathrm{a}\mathrm{s}\mathrm{a}\mathrm{l}\:\mathrm{l}\mathrm{e}\mathrm{n}\mathrm{g}\mathrm{t}\mathrm{h}\:\left(\mathrm{P}3\right)}\:\times\:100$
I6	Index 3	$\:\frac{\mathrm{g}\mathrm{r}\mathrm{e}\mathrm{a}\mathrm{t}\mathrm{e}\mathrm{s}\mathrm{t}\:\mathrm{n}\mathrm{e}\mathrm{u}\mathrm{r}\mathrm{o}\mathrm{c}\mathrm{r}\mathrm{a}\mathrm{n}\mathrm{i}\mathrm{u}\mathrm{m}\:\mathrm{b}\mathrm{r}\mathrm{e}\mathrm{a}\mathrm{d}\mathrm{t}\mathrm{h}\:\left(\mathrm{P}31\right)}{\mathrm{c}\mathrm{o}\mathrm{n}\mathrm{d}\mathrm{y}\mathrm{l}\mathrm{o}\mathrm{b}\mathrm{a}\mathrm{s}\mathrm{a}\mathrm{l}\:\mathrm{l}\mathrm{e}\mathrm{n}\mathrm{g}\mathrm{t}\mathrm{h}\:\left(\mathrm{P}2\right)}\:\times\:100$
I7	Foramen magnum index	$\:\frac{\mathrm{h}\mathrm{e}\mathrm{i}\mathrm{g}\mathrm{h}\mathrm{t}\:\mathrm{o}\mathrm{f}\:\mathrm{t}\mathrm{h}\mathrm{e}\:\mathrm{f}\mathrm{o}\mathrm{r}\mathrm{a}\mathrm{m}\mathrm{e}\mathrm{n}\:\mathrm{m}\mathrm{a}\mathrm{g}\mathrm{n}\mathrm{u}\mathrm{m}\:\left(\mathrm{P}30\right)}{\mathrm{g}\mathrm{r}\mathrm{e}\mathrm{a}\mathrm{t}\mathrm{e}\mathrm{s}\mathrm{t}\:\mathrm{b}\mathrm{r}\mathrm{e}\mathrm{a}\mathrm{d}\mathrm{t}\mathrm{h}\:\mathrm{o}\mathrm{f}\:\mathrm{t}\mathrm{h}\mathrm{e}\:\mathrm{f}\mathrm{o}\mathrm{r}\mathrm{a}\mathrm{m}\mathrm{e}\mathrm{n}\:\mathrm{m}\mathrm{a}\mathrm{g}\mathrm{n}\mathrm{u}\mathrm{m}\:\left(\mathrm{P}29\right)}\:\times\:100$

*I* Index, *P* Parameter

## Multivariate morphometric analysis

The interspecific morphometric variation was explored using Principal Component Analysis (PCA) [40]. Separate PCA were performed on three datasets consisting of selected, measured parameters, with each parameter represented by the individual specimen mean (final metric): (1) skull measurements scaled by dividing the total cranial length (Parameter 1), (2) mandible measurements scaled by dividing the total mandibular length (Parameter 45) and (3) calculated shape indices. The choice of parameters included in the PCA was driven by the incompleteness of the collected skull specimens (a valuable and limited research material). The PCA was executed using *ade4* [41, 42] and *factoextra* [43] libraries in R [44]. The first two principal components (PC1 and PC2) were retained for interpretation. Descriptive statistics (mean, Min and Max) for absolute and index values are presented in Table 4.

**Table 4:** Calculated statistics for the Aardwolf (*n* = 3), the spotted hyena (*n* = 5), the striped hyena (*n* = 1) and the brown hyena (*n* = 3). All parameters are expressed in mm. Indices are unitless

Parameter / Index	Species	Mean	Min	Max
P1	Aardwolf	129.00	123.98	134.14
	Spotted hyena	255.86	237.77	270.00
	Striped hyena	237.50	–	–
	Brown hyena	264.98	259.93	275.00
P2	Aardwolf	127.39	121.80	133.51
	Spotted hyena	240.14	227.98	255.00
	Striped hyena	216.07	–	–
	Brown hyena	233.97	225.00	240.00
P3	Aardwolf	121.21	117.30	126.28
	Spotted hyena	223.06	206.18	235.00
	Striped hyena	205.12	–	–
	Brown hyena	220.50	215.00	225.00
P4	Aardwolf	35.18	31.12	37.84
	Spotted hyena	69.28	68.21	70.35
	Striped hyena	–	–	–
	Brown hyena	72.59	64.60	88.18
P5	Aardwolf	86.13	81.15	91.58
	Spotted hyena	159.93	151.87	168.00
	Striped hyena	–	–	–
	Brown hyena	152.59	149.00	157.18
P6	Aardwolf	65.03	63.36	66.03
	Spotted hyena	146.90	137.50	159.80
	Striped hyena	132.97	–	–
	Brown hyena	148.50	145.87	153.51
P7	Aardwolf	54.93	53.20	58.40
	Spotted hyena	100.07	97.05	101.93
	Striped hyena	87.98	–	–
	Brown hyena	98.32	89.56	103.84
P8	Aardwolf	71.65	68.02	76.72
	Spotted hyena	132.53	123.90	138.45
	Striped hyena	115.57	–	–
	Brown hyena	129.16	123.77	134.68
P9	Aardwolf	43.63	40.52	48.01
	Spotted hyena	56.68	52.20	63.07
	Striped hyena	57.22	–	–
	Brown hyena	59.85	52.21	65.00
P10	Aardwolf	63.56	62.00	64.67
	Spotted hyena	113.34	104.07	118.00
	Striped hyena	98.38	–	–
	Brown hyena	103.23	96.00	110.00
P11	Aardwolf	50.02	44.67	53.14
	Spotted hyena	87.48	78.77	98.28
	Striped hyena	79.60	–	–
	Brown hyena	92.65	86.34	99.68
P12	Aardwolf	65.04	63.23	68.32
	Spotted hyena	120.88	107.80	137.25
	Striped hyena	113.70	–	–
	Brown hyena	118.65	109.94	127.10
P12a	Aardwolf	64.62	62.42	68.22
	Spotted hyena	127.79	120.76	134.81
	Striped hyena	113.70	–	–
	Brown hyena	117.83	108.80	126.80
P13	Aardwolf	23.65	22.62	25.45
	Spotted hyena	47.96	44.22	54.34
	Striped hyena	41.50	–	–
	Brown hyena	34.83	30.97	39.88
P13a	Aardwolf	23.16	22.06	24.86
	Spotted hyena	49.44	45.33	53.55
	Striped hyena	41.50	–	–
	Brown hyena	33.79	29.76	38.71
P14	Aardwolf	26.74	24.78	28.20
	Spotted hyena	82.61	79.73	86.39
	Striped hyena	–	–	–
	Brown hyena	78.05	75.50	80.07
P15	Aardwolf	38.60	36.44	41.53
	Spotted hyena	105.11	99.56	115.48
	Striped hyena	86.48	–	–
	Brown hyena	98.07	94.75	102.92
P16	Aardwolf	2.56	–	–
	Spotted hyena	3.58	–	–
	Striped hyena	4.43	–	–
	Brown hyena	5.45	4.66	6.23
P17	Aardwolf	18.28	–	–
	Spotted hyena	79.26	76.83	84.99
	Striped hyena	–	–	–
	Brown hyena	73.64	71.66	75.96
P18	Aardwolf	N/A	N/A	N/A
	Spotted hyena	36.75	35.15	38.68
	Striped hyena	26.81	–	–
	Brown hyena	34.34	32.71	36.32
P19	Aardwolf	N/A	N/A	N/A
	Spotted hyena	20.61	19.80	21.38
	Striped hyena	16.25	–	–
	Brown hyena	21.47	20.22	22.30
P20	Aardwolf	N/A	N/A	N/A
	Spotted hyena	35.24	34.47	36.47
	Striped hyena	26.94	–	–
	Brown hyena	32.59	29.90	36.11
P21	Aardwolf	2.56	–	–
	Spotted hyena	–	–	–
	Striped hyena	5.07	–	–
	Brown hyena	10.32	5.66	13.60
P22	Aardwolf	1.74	–	–
	Spotted hyena	–	–	–
	Striped hyena	12.78	–	–
	Brown hyena	7.84	4.80	12.43
P23	Aardwolf	22.21	16.42	28.01
	Spotted hyena	41.43	36.13	46.58
	Striped hyena	32.88	–	–
	Brown hyena	38.73	35.93	41.25
P24	Aardwolf	14.47	14.40	14.55
	Spotted hyena	23.19	22.15	23.76
	Striped hyena	17.82	–	–
	Brown hyena	19.10	17.80	19.87
P25	Aardwolf	46.58	46.10	47.06
	Spotted hyena	99.98	89.92	116.48
	Striped hyena	81.87	–	–
	Brown hyena	90.55	86.00	93.60
P26	Aardwolf	39.21	38.97	39.45
	Spotted hyena	86.29	79.25	98.11
	Striped hyena	69.38	–	–
	Brown hyena	72.69	71.80	73.58
P27	Aardwolf	26.48	26.38	26.58
	Spotted hyena	50.73	49.32	51.90
	Striped hyena	40.95	–	–
	Brown hyena	49.45	49.08	50.02
P28	Aardwolf	42.63	41.62	43.63
	Spotted hyena	82.61	76.47	87.85
	Striped hyena	70.43	–	–
	Brown hyena	78.18	75.81	81.45
P29	Aardwolf	14.83	14.20	16.08
	Spotted hyena	23.92	21.93	25.53
	Striped hyena	15.18	–	–
	Brown hyena	20.93	20.00	22.17
P30	Aardwolf	10.37	9.18	12.42
	Spotted hyena	21.09	18.52	24.81
	Striped hyena	20.00	–	–
	Brown hyena	19.86	16.90	22.00
P31	Aardwolf	44.41	44.00	45.00
	Spotted hyena	76.51	71.98	81.30
	Striped hyena	65.28	–	–
	Brown hyena	62.57	60.00	67.72
P32	Aardwolf	70.84	67.62	74.07
	Spotted hyena	171.02	161.33	183.00
	Striped hyena	156.12	–	–
	Brown hyena	176.97	165.00	185.90
P33	Aardwolf	31.58	30.90	32.68
	Spotted hyena	44.75	40.47	50.09
	Striped hyena	37.58	–	–
	Brown hyena	42.19	39.32	44.08
P34	Aardwolf	51.27	46.28	57.56
	Spotted hyena	78.91	70.85	83.27
	Striped hyena	84.57	–	–
	Brown hyena	83.36	75.40	91.72
P35	Aardwolf	27.67	26.30	30.00
	Spotted hyena	64.50	61.08	70.24
	Striped hyena	50.27	–	–
	Brown hyena	59.44	51.44	68.48
P36	Aardwolf	37.08	36.63	37.43
	Spotted hyena	105.75	97.13	114.83
	Striped hyena	89.06	–	–
	Brown hyena	76.72	70.21	89.08
P37	Aardwolf	34.76	32.72	36.69
	Spotted hyena	60.44	56.77	65.87
	Striped hyena	43.58	–	–
	Brown hyena	56.73	53.98	62.06
P38	Aardwolf	34.87	33.17	37.11
	Spotted hyena	61.95	58.64	64.31
	Striped hyena	46.93	–	–
	Brown hyena	56.80	54.06	59.04
P39	Aardwolf	23.66	21.30	25.66
	Spotted hyena	44.84	42.39	48.11
	Striped hyena	38.70	–	–
	Brown hyena	36.06	33.02	40.78
P40	Aardwolf	24.58	23.68	25.15
	Spotted hyena	39.21	36.67	43.37
	Striped hyena	39.14	–	–
	Brown hyena	33.20	27.55	38.54
P41	Aardwolf	30.77	29.32	32.54
	Spotted hyena	61.96	56.77	70.48
	Striped hyena	46.13	–	–
	Brown hyena	53.71	51.45	57.61
P42	Aardwolf	36.98	34.62	38.28
	Spotted hyena	95.82	91.98	97.30
	Striped hyena	91.97	–	–
	Brown hyena	89.12	87.52	92.27
P43	Aardwolf	31.57	31.00	32.33
	Spotted hyena	73.43	63.55	78.42
	Striped hyena	64.53	–	–
	Brown hyena	64.83	55.65	71.45
P44	Aardwolf	88.92	88.10	89.61
	Spotted hyena	180.01	167.68	191.50
	Striped hyena	163.82	–	–
	Brown hyena	185.51	180.00	190.00
P45	Aardwolf	92.03	88.89	95.30
	Spotted hyena	185.08	171.41	196.00
	Striped hyena	172.51	–	–
	Brown hyena	184.65	181.50	187.50
P46	Aardwolf	87.91	86.29	90.88
	Spotted hyena	174.90	163.45	185.00
	Striped hyena	157.05	–	–
	Brown hyena	175.51	171.50	180.00
P47	Aardwolf	76.59	75.00	78.71
	Spotted hyena	157.22	145.23	166.50
	Striped hyena	143.00	–	–
	Brown hyena	161.01	155.00	167.50
P48	Aardwolf	74.60	72.58	77.91
	Spotted hyena	150.95	141.15	158.22
	Striped hyena	157.02	–	–
	Brown hyena	149.35	145.00	153.33
P49	Aardwolf	76.86	74.69	79.19
	Spotted hyena	160.65	145.10	170.00
	Striped hyena	172.77	–	–
	Brown hyena	159.74	156.00	162.50
P50	Aardwolf	38.96	38.92	39.01
	Spotted hyena	92.91	87.05	98.22
	Striped hyena	80.17	–	–
	Brown hyena	90.32	87.89	94.57
P51	Aardwolf	24.73	–	–
	Spotted hyena	83.58	78.64	91.85
	Striped hyena	65.22	–	–
	Brown hyena	77.75	76.21	80.17
P52	Aardwolf	22.67	–	–
	Spotted hyena	69.85	68.77	71.22
	Striped hyena	53.60	–	–
	Brown hyena	64.99	62.76	68.65
P53	Aardwolf	1.95	–	–
	Spotted hyena	28.11	25.86	30.10
	Striped hyena	18.43	–	–
	Brown hyena	23.37	22.78	24.48
P54	Aardwolf	10.97	–	–
	Spotted hyena	55.14	52.45	57.04
	Striped hyena	45.35	–	–
	Brown hyena	56.19	54.85	58.02
P55	Aardwolf	8.58	–	–
	Spotted hyena	42.49	42.09	43.17
	Striped hyena	34.18	–	–
	Brown hyena	42.75	41.06	44.80
P56	Aardwolf	N/A	N/A	N/A
	Spotted hyena	27.62	26.43	29.16
	Striped hyena	19.36	–	–
	Brown hyena	23.76	23.07	24.94
P57	Aardwolf	N/A	N/A	N/A
	Spotted hyena	12.17	12.02	12.38
	Striped hyena	10.14	–	–
	Brown hyena	12.36	11.79	12.83
P58	Aardwolf	N/A	N/A	N/A
	Spotted hyena	28.27	25.86	30.07
	Striped hyena	18.43	–	–
	Brown hyena	23.22	22.75	24.15
P59	Aardwolf	5.32	4.62	6.64
	Spotted hyena	13.72	12.75	15.36
	Striped hyena	13.62	–	–
	Brown hyena	14.06	13.17	15.48
P60	Aardwolf	29.00	26.19	32.33
	Spotted hyena	82.08	80.13	86.90
	Striped hyena	69.23	–	–
	Brown hyena	82.07	79.64	83.45
P61	Aardwolf	14.96	13.78	17.00
	Spotted hyena	45.91	40.81	50.10
	Striped hyena	40.56	–	–
	Brown hyena	42.85	40.24	45.41
P62	Aardwolf	14.57	13.42	15.43
	Spotted hyena	31.41	28.98	34.81
	Striped hyena	30.88	–	–
	Brown hyena	36.09	33.62	38.81
I1	Aardwolf	56.01	54.54	57.48
	Spotted hyena	67.03	61.30	73.20
	Striped hyena	65.73	–	–
	Brown hyena	66.81	63.46	71.52
I2	Aardwolf	133.16	127.11	139.22
	Spotted hyena	166.74	159.36	174.07
	Striped hyena	177.44	–	–
	Brown hyena	180.20	173.34	184.23
I3	Aardwolf	85.42	77.43	92.37
	Spotted hyena	185.56	154.01	217.53
	Striped hyena	155.65	-	-
	Brown hyena	128.41	113.69	137.05
I4	Aardwolf	34.47	32.80	36.30
	Spotted hyena	29.97	27.35	32.52
	Striped hyena	27.49	–	–
	Brown hyena	23.65	21.82	26.05
I5	Aardwolf	36.68	34.84	38.36
	Spotted hyena	34.34	32.35	36.67
	Striped hyena	31.83	–	–
	Brown hyena	28.38	26.67	30.57
I6	Aardwolf	34.92	32.96	36.95
	Spotted hyena	31.88	30.25	33.88
	Striped hyena	30.21	–	–
	Brown hyena	26.75	25.00	28.58
I7	Aardwolf	69.60	64.65	77.20
	Spotted hyena	88.14	78.68	100.81
	Striped hyena	105.77	–	–
	Brown hyena	94.68	84.50	100.32

*I* Index, *Max* Maximum, *Min* Minimum, *N/A* Not applicable, *P* Parameter

*Note that min and max could not be presented for the striped hyena due to the availability of only one skull(*n = 1).*

## Results

### Morphological differences and similarities of the cranial bones

**Fig. 4:**
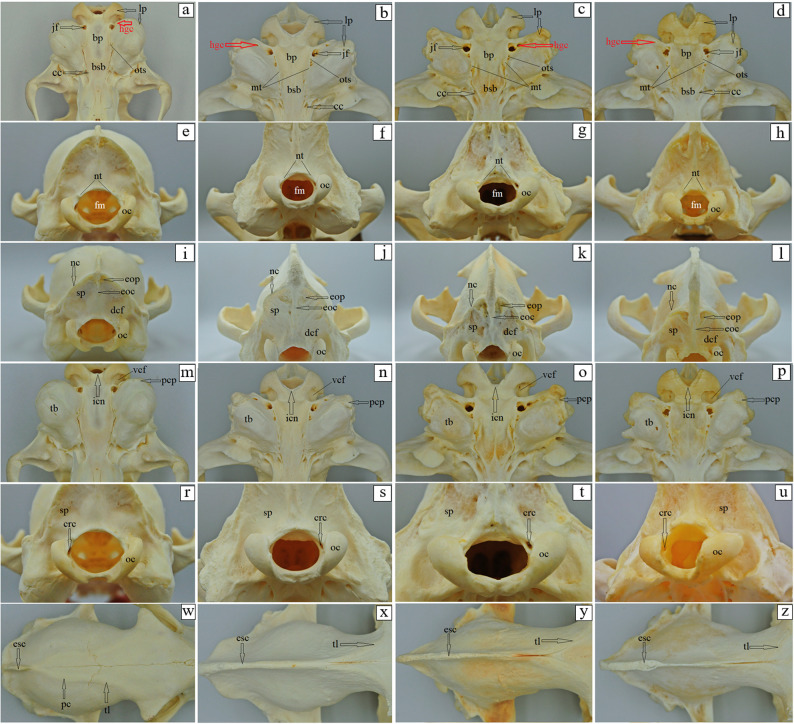
Occipital bone anatomy of the aardwolf (**a**, **e**, **i**, **m**, **r**, **w**), the spotted hyena (**b**, **f**, **j**, **n**, **s**, **x**), the brown hyena (**c**, **g**, **k**, **o**, **t**, **y**) and the striped hyena (**d**, **h**, **l**, **p**, **u**, **z**). bsb – basisphenoid bone, bp – basilar part of the occipital bone, cc – carotic canal (black arrow), crc – condylar canal (black arrow), dcf – dorsal condylar fosse, eoc – external occipital crest (black arrow), eop – external occipital protuberance (black arrow), esc – external sagittal crest (black arrow), fm – foramen magnum, hgc – hypoglossal canal (red arrow), icn – intercondylar noch (black arrow), jf – jugular foramen (black arrow), lp – lateral part of the occipital bone (black arrow), mt – muscular tubercule, nc – nuchal crest (black arrow), nt – nuchal tubercule, oc – occipital condyli, ots – occipitotympanic suture, pc – parietal crest (black arrow), pcp – paracondylar process (black arrow), sp – squamous part of the occipital bone, tb – tympanic bulla, tl – temporal line (black arrow), vcf – ventral condylar fosse

**Fig. 5:**
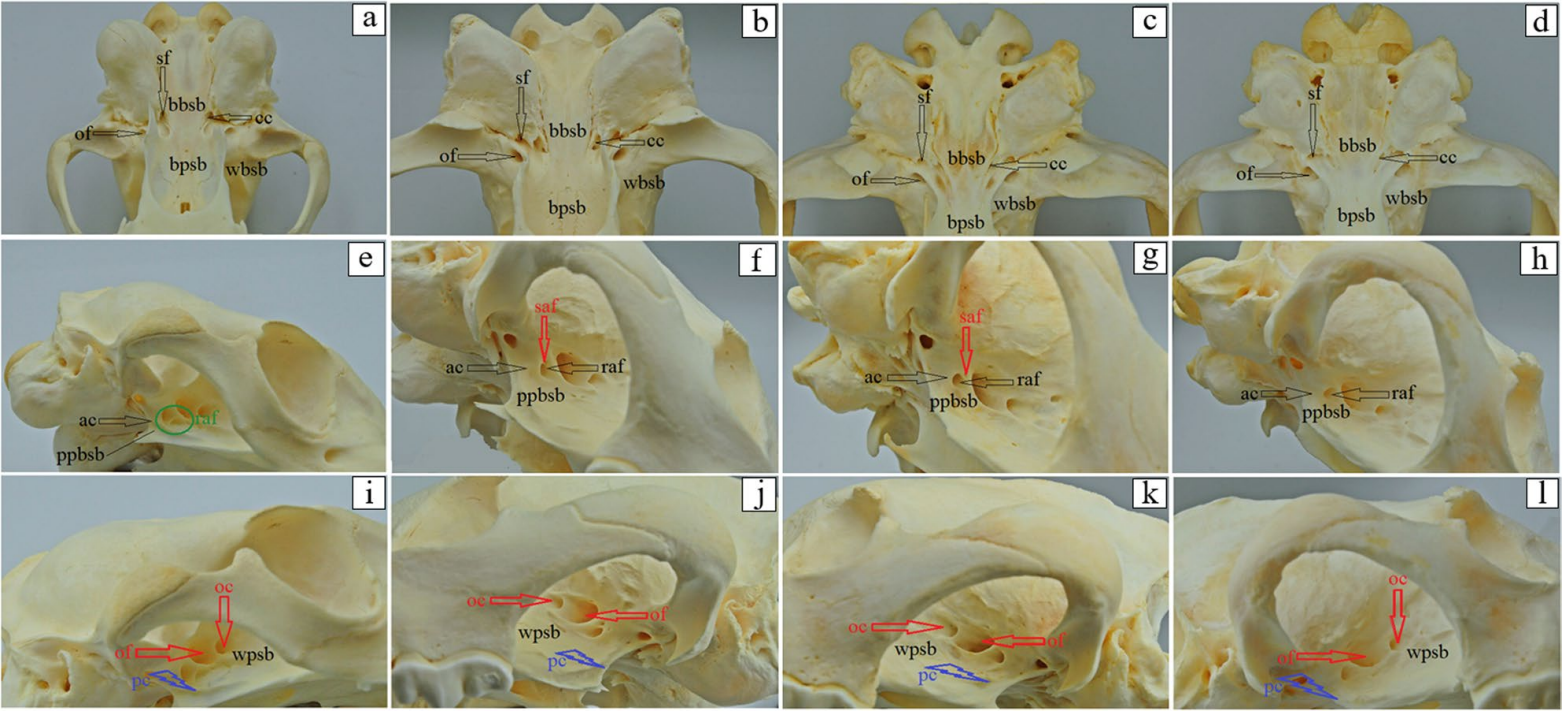
Basisphenoid and presphenoid bones anatomy of the aardwolf (**a**, **e**, **i**), the spotted hyena (**b**, **f**, **j**), the brown hyena (**c**, **g**, **k**) and the striped hyena (**d**, **h**, **l**). ac – alar (pterygoid) canal (black arrow), bbsb – body of the basisphenoid bone, bpsb – body of the presphenoid bone, cc – carotid canal (black arrow), oc – optic canal (red arrow), of – orbital fissure (red arrow), of – oval foramen (black arrow), pc – pterygoid crest (blue bolt symbol), ppbsb – pterygoid process of the basisphenoid bone, raf – rostral alar foramen (black arrow – in the spotted hyena, brown hyena and striped hyena), raf – rostral alar foramen (green circular marker – in the aardwolf), saf – small alar foramen (red arrow), sf – spinosus foramen (black arrow), wbsb– wings of basisphenoid bone, wpsb – wings of the presphenoid bone

**Fig. 6:**
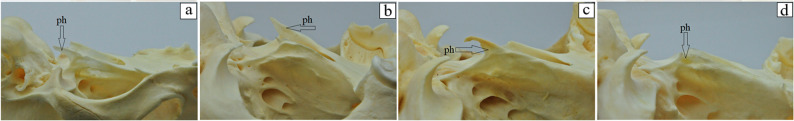
Pterygoid bone anatomy of the aardwolf (**a**), the spotted hyena (**b**), the brown hyena (**c**), and the striped hyena (**d**). ph – pterygoid hamulus (black arrow)

**Fig. 7:**
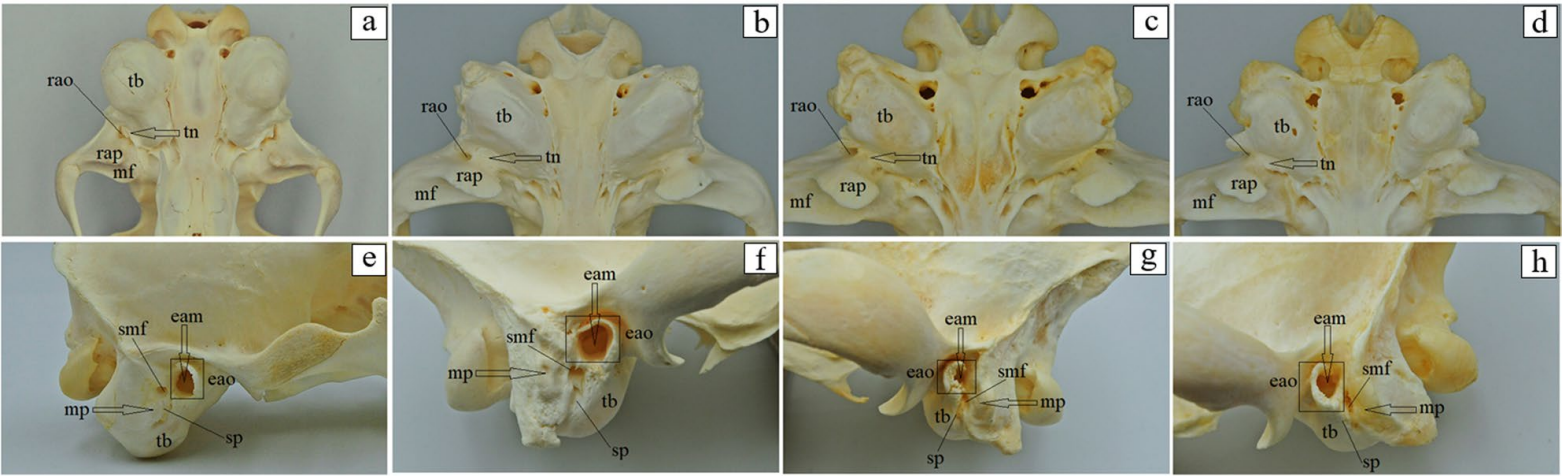
Temporal bone anatomy of the aardwolf (**a**, **e**), the spotted hyena (**b**, **f**), the brown hyena (**c**, **g**), and the striped hyena (**d**, **h**). eam – external acoustic meatus (black arrow), eao – external acoustic opening (black square marker), mf – mandibular fossa, mp – mastoid process (black arrow), rao – retroarticular opening, rap – retroarticular process, smf – stylomastoid foramen, sp – styloid process, tb – tympanic bulla, tn – tympanic notch (black arrow)

**Fig. 8:**
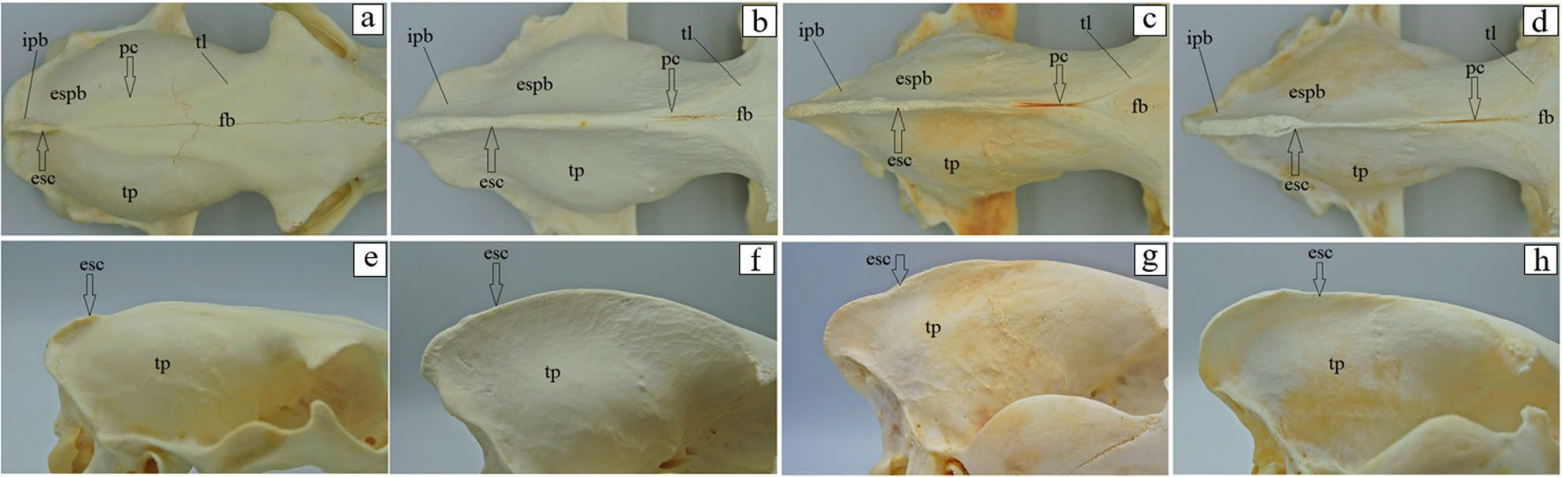
Parietal bone anatomy of the aardwolf (**a**, **e**), the spotted hyena (**b**, **f**), the brown hyena (**c**, **g**), and the striped hyena (**d**, **h**). esc – external sagittal crest (black arrow), espb – external surface of the parietal bone, fb – frontal bone, ipb – interparietal bone, pc – parietal crest (black arrow), tl – temporal line, tp – temporal plane

**Fig. 9:**
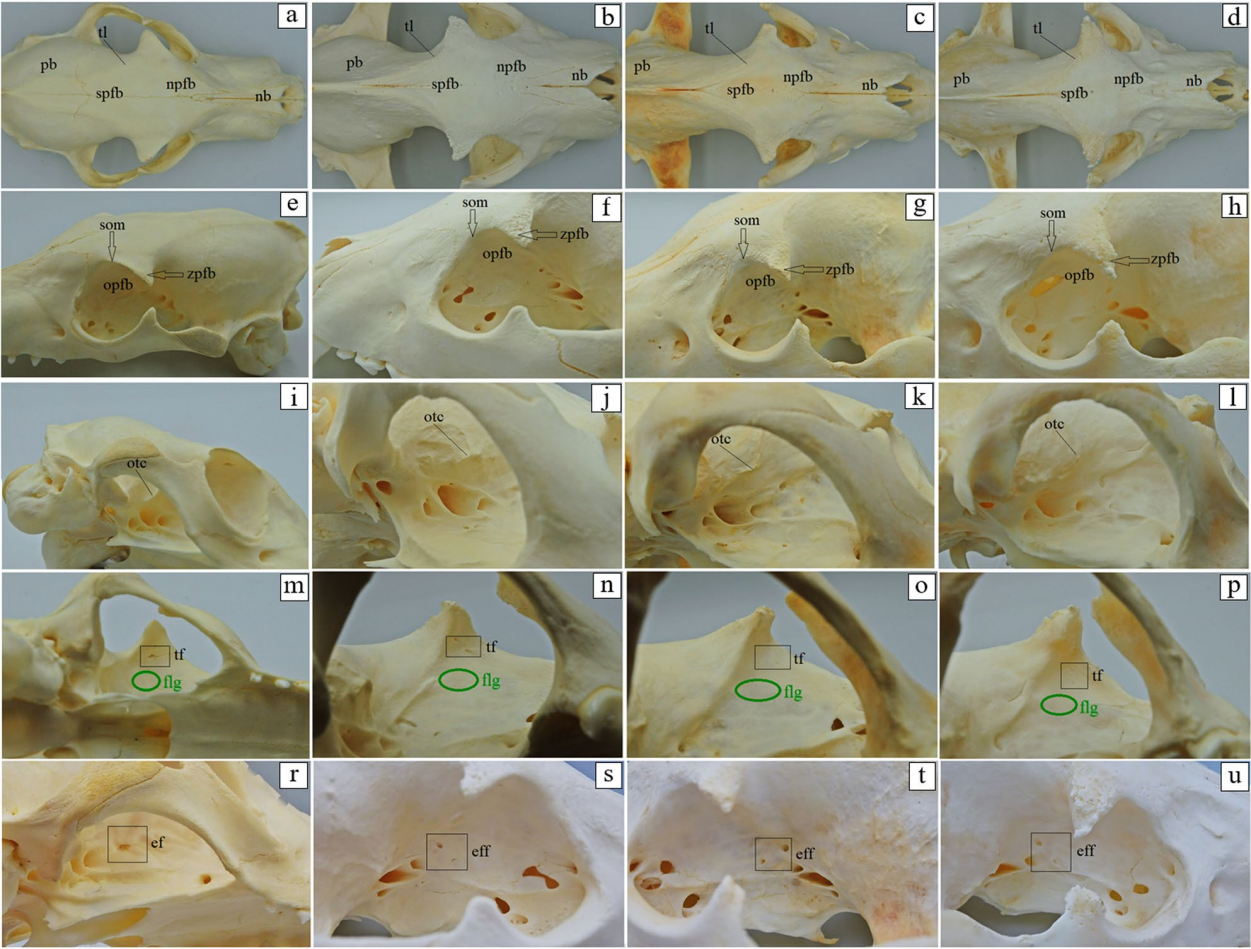
Frontal bone anatomy of the aardwolf (**a**, **e**, **i**, **m**, **r**), the spotted hyena (**b**, **f**, **j**, **n**, **s**), the brown hyena (**c**, **g**, **k**, **o**, **t**), and the striped hyena (**d**, **h**, **l**, **p**, **u**). ef – ethmoid foramen (black square marker), eff – ethmoid foramina (black square marker), flg – fossa for lacrimal gland (green oval marker), nb – nasal bone, npfb – nasal part of frontal bone, otc – orbitotemporal crest, opfb – orbital part of the frontal bone, pb – parietal bone, som – supraorbital margin (black arrow), spfb – squamous part of the frontal bone, tf – trochlear fovea (black square marker), tl – temporal line, zpfb – zygomatic process of the frontal bone (black arrow)

The basilar part of the occipital bone of the aardwolf is shaped like a broad cylinder, similarly as in the brown hyena and the striped hyena, while in the spotted hyena it takes the form of a pyramid, constituting the base of the skull (Fig. 4a-d). The lateral border of the basilar part does not form a foramen lacerum; instead, a small jugular foramen is present in its place in both the aardwolf and the other three species (Fig. 4a-d). Immediately on the occipitotympanic suture is a wide hypoglossal canal (Fig. 4a-d). Anterior to the hypoglossal canal, a small round carotid canal is visible in all four species (Fig. 4a-d). On the external surface of the basilar part, at the junction with the basisphenoid bone, no muscular tubercles are visible in the aardwolf (Fig. 4a), while two well-defined tubercles are present in the spotted hyena, whereas in the brown hyena and the striped hyena, these structures appear as two small prominences (Fig. 4b-d). Above the foramen magnum, two well-defined flat nuchal tubercles are present, projecting beyond its margin in the aardwolf and the striped hyena (Fig. 4e and h), while in the spotted hyena and the brown hyena (Fig. 4f and g) they are reduced and do not extend beyond the edge of the foramen. Immediately above the occipital condyles, deep dorsal condylar fossae are present (Fig. 4i-l). Between the occipital condyles and the paracondylar processes, deep ventral condylar fossae are observed (Fig. 4m and p). Laterally to the occipital condyles, the paracondylar processes fuse with the tympanic bulla, forming very faint elevations in the aardwolf (Fig. 4m). In contrast, in the spotted hyena, brown hyena, and striped hyena the processes are present as distinct, prominent, and thickened free bony projections extending ventrally (Fig. 4n-p). Along their ventral margin, a wide intercondylar notch is present in the aardwolf, a narrow notch in the spotted hyena, and no notch in the brown hyena and striped hyena (Fig. 4m-p). On the internal surface of the occipital condyles, the entrance to the condylar canal is visible (Fig. 4r-u). The squamous part of the occipital bone features a sharp nuchal crest, which extends laterally into the temporal line and continues sagittally into a prominent and high external sagittal crest (Fig. 4i and l). In the aardwolf, the external occipital crest is sharp and low only in its upper portion (Fig. 4i), while in the spotted hyena, brown hyena and striped hyena, the external occipital crest is high and sharply defined along its entire length (Fig. 4j-l). The external occipital protuberance is faintly visible in the aardwolf (Fig. 4w), while in the spotted hyena, brown hyena and striped hyena is well-defined (Fig. 4x-z). The evaluation of the basisphenoid bone reveals that in the aardwolf and other three hyena species, the lacerum foramen is absent; instead, a carotid canal is present, located within the tympanic part of the temporal bone. In addition, a rostrally placed oval foramen is observed and, laterally to it, a spinosus foramen (Fig. 5a-d). In the rostral part of the basisphenoid bone, the pterygoid process expands, containing a horizontally oriented alar (pterygoid) canal with a rostral alar foramen and a caudal alar foramen (Fig. 5e-h). In the aardwolf, the rostral alar foramen is double (Fig. 5e). Furthermore, in the spotted hyena and brown hyena, a small alar foramen is present on the right side (Fig. 5f and g). Evaluation of presphenoid bone reveals that the wings are well developed in all four species. From a superior view, the optic canal is distinguishable, located directly above the orbital fissure (Fig. 5i-l). The pterygoid crest is poorly developed (Fig. 5i-l). The free end of the pterygoid bone, known as the pterygoid hamulus is well developed in the aardwolf, spotted hyena and brown hyena, while it is less developed in the striped hyena (Fig. 6a-d). Caudal to the mandibular fossa, a short and straight retroarticular process is present in the aardwolf (Fig. 7a). In contrast, in the spotted hyena, the brown hyena and the striped hyena, the retroarticular process is strongly curved inward (Fig. 7b-d). The retroarticular opening is present only in the aardwolf, while in the other three species, a deep tympanic notch is observed (Fig. 7a-d). The tympanic part of the temporal bone has a highly inflated tympanic bulla, which encloses the tympanic cavity, which is accessed through the external acoustic meatus, resulting in a large external acoustic opening (Fig. 7e-h). The muscular process is absent in all examined species. On the ventral surface of the petrous part of the temporal bone, a poorly developed mastoid process and a styloid process are observed (Fig. 7e-h). Between the styloid process and the tympanic part of the temporal bone, the stylomastoid foramen is clearly visible (Fig. 7e-h). The external surface of the parietal bone is convex in all examined species. In the aardwolf, the external sagittal crest is very short and low, restricted to the interparietal bone (Fig. 8a). However, in the spotted hyena, the brown hyena, and the striped hyena, the external sagittal crest is very long and well-developed. This crest extends rostrally to the frontal bone, continuing as the temporal line (Fig. 8b-d). In the aardwolf, the entire temporal plane is strongly concave. Moving forward, it bifurcates into two long and broad parietal crests, which extend toward the frontal bone as the temporal line (Fig. 8e). In contrast, in the spotted hyena, brown hyena, and striped hyena, the temporal plane is very flattened in its upper part and became convex toward the lower region (Fig. 8f-h). The squamous part of the frontal bone is separated by a sharp supraorbital margin in the aardwolf (Fig. 9e), whereas in the spotted hyena, brown hyena, and striped hyena the supraorbital margin is blunt (Fig. 9f-h). The temporal line is only faintly marked in the aardwolf and the brown hyena (Fig. 9a and c), whereas it is well defined in the spotted hyena and striped hyena (Fig. 9b and d). The supraorbital foramen is absent. The short zygomatic process of the frontal bone, together with the frontal process of the zygomatic bone, forms an open-type orbit (Fig. 9e-h). The orbitotemporal crest is weakly defined in all examined species (Fig. 9i-l). The fossa for the lacrimal gland is well developed in the aardwolf and striped hyena (Fig. 9m and p). The nasal part of the frontal bone forms a single-plane extension of the squamous part of the frontal bone, collectively known as the nasofrontal part (Fig. 9a-d). The orbital part of the frontal bone is characterized by a well-defined trochlear fovea, present in all specimens studied (Fig. 9m-p). On the orbital surface, a single foramen is observed in the aardwolf (Fig. 9r), whereas two ethmoid foramina are present in the spotted hyena, brown hyena, and striped hyena (Fig. 9s-u).

### Morphological differences and similarities of the facial bones

**Fig. 10:**
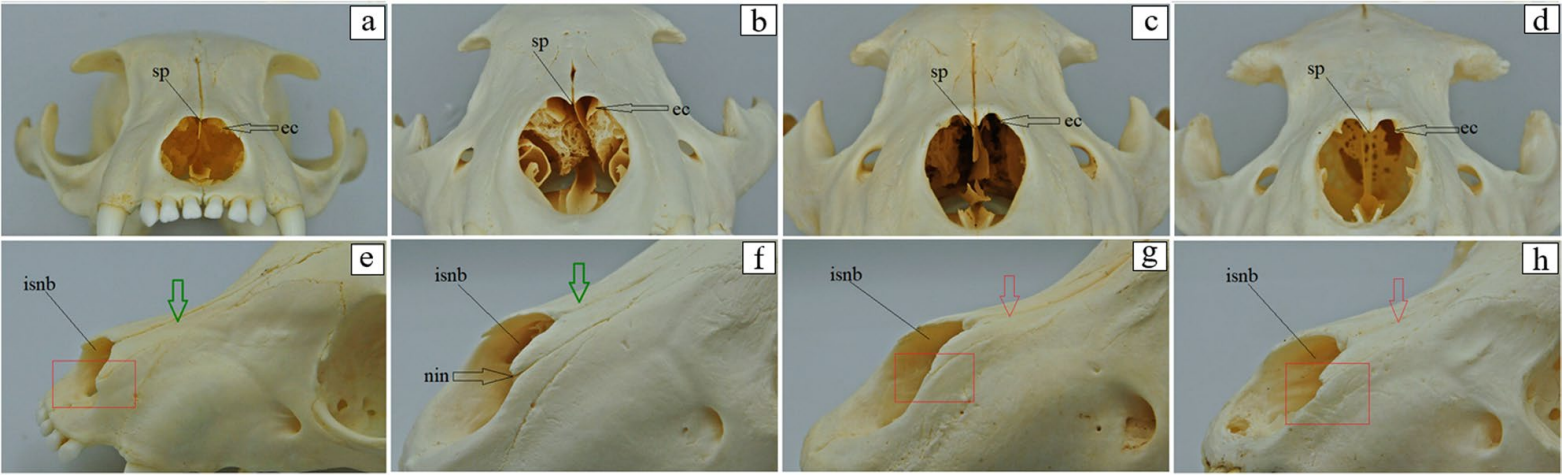
Nasal bone anatomy of the aardwolf (**a**, **e**), the spotted hyena (**b**, **f**), the brown hyena (**c**, **g**), and the striped hyena (**d**, **h**). ec – ethmoid crest (black arrow), isnb – internal surface of the nasal bone, nin – nasoincisive notch (black arrow in the spotted hyena and red square marker indicating the absence of the nasoincisive notch in the aardwolf, brown hyena and the striped hyena), sp – septal process, green arrow – concave external surface of the nasal bone in the aardwolf and the spotted hyena, red arrow – more concave external surface of the nasal bone in the brown and the striped hyenas

**Fig. 11:**
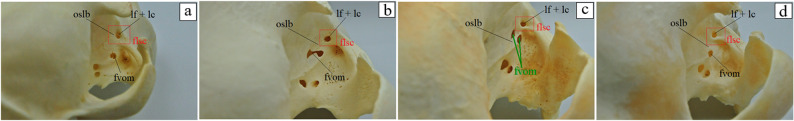
Lacrimal bone anatomy of the aardwolf (**a**), the spotted hyena (**b**), the brown hyena (**c**), and the striped hyena (**d**). flsc – fossa for lacrimal sac (red square), fvom – foramen for ventral oblique muscle attachment (green – indicates two openings in the brown hyena), lf + lc – lacrimal foramen and lacrimal canal, oslb – orbital surface of the lacrimal bone

**Fig. 12:**
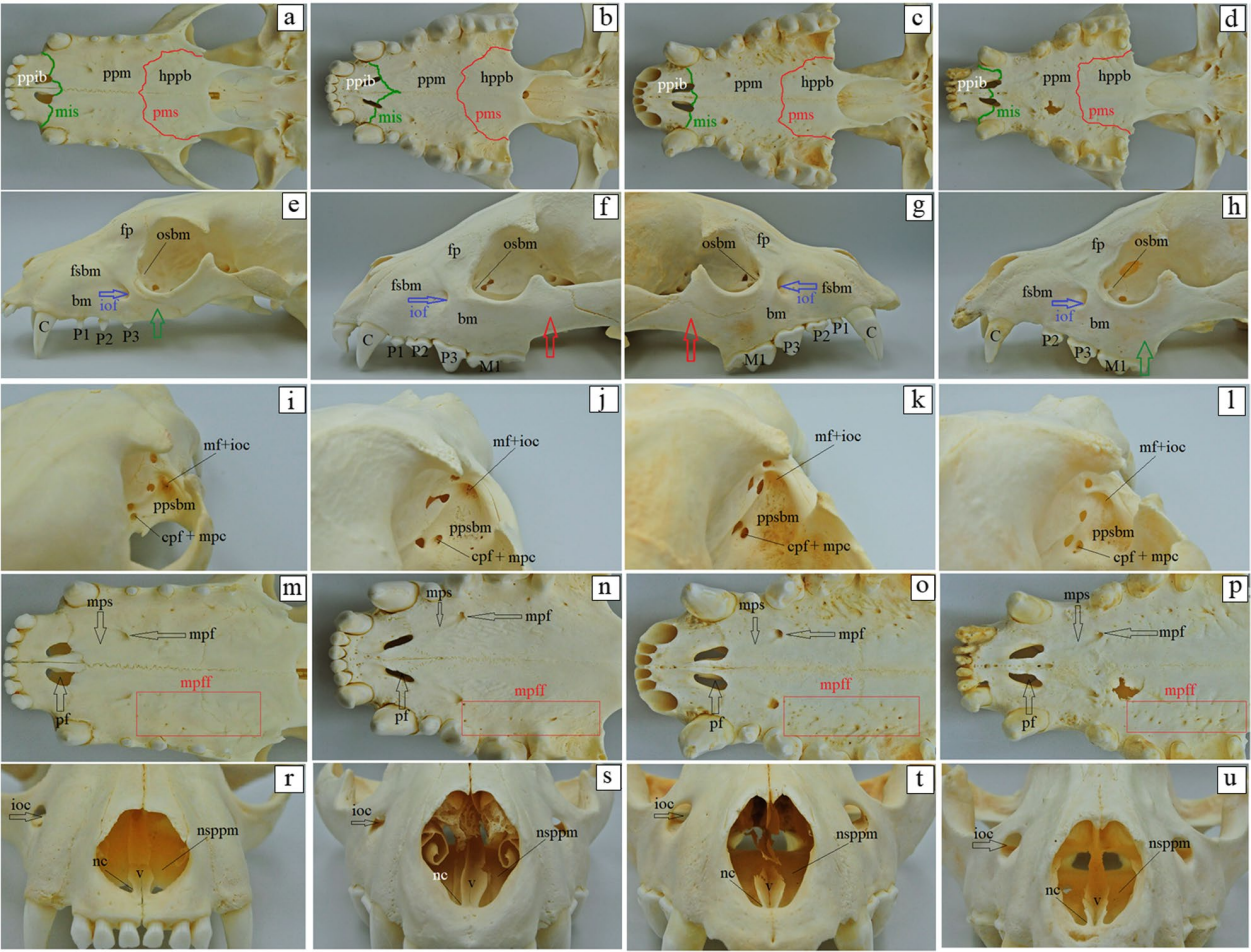
Maxilla anatomy of the aardwolf (**a**, **e**, **i**, **m**), the spotted hyena (**b**, **f**, **j**, **n**), the brown hyena (**c**, **g**, **k**, **o**), and the striped hyena (**d**, **h**, **l**, **p**). bm – body of the maxilla, C – canine, cpf + mpc – caudal palatine foramen and major palatine canal, fb – frontal bone, fsbm – facial surface of the body of maxilla, hppb – horizontal plate of the palatine bone, iof – infraorbital foramen (blue arrow), mf+ioc – maxillary foramen with infraorbital canal, mis – maxilloincisive suture, mpf – major palatine foramen (black arrow), mpff – minor palatine foramina (red square marker), mps – major palatine sulcus (black arrow), M1 – first molar, osbm – orbital surface of the body of maxilla, pf – palatine fissure (black arrow), pms – palatomaxillary suture, ppib – palatine process of the incisive bone, ppm – palatine process of the maxilla, ppsbm – pterygopalatine surface of the body of maxilla, P1 – first premolar, P2 – second premolar, P3 – third premolar, red arrow – indicates the contribution of the mandible to the formation of the zygomatic arch in the spotted hyena and the brown hyena, green arrow – indicates the absence of mandibular contribution to the formation of the zygomatic arch in the aardwolf and the striped hyena

**Fig. 13:**
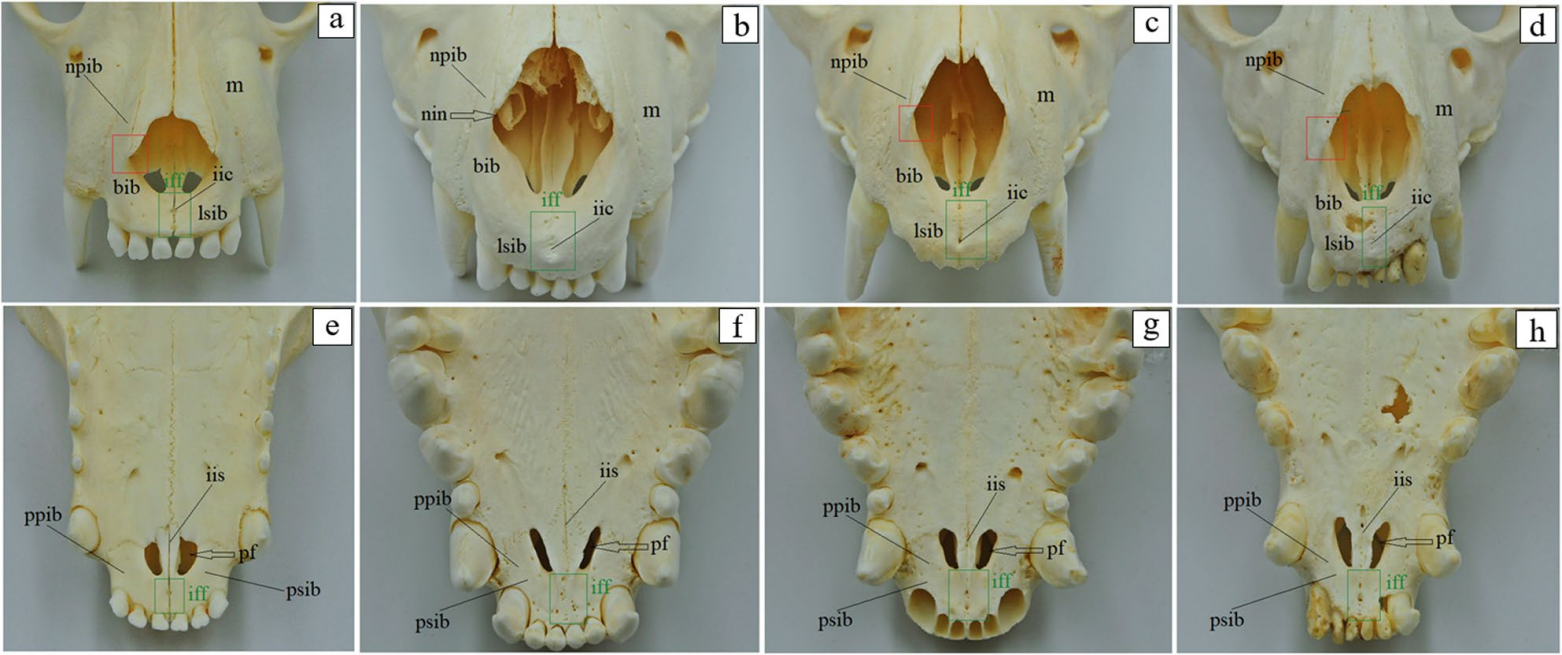
Incisive bone anatomy of the aardwolf (**a**, **e**), the spotted hyena (**b**, **f**), the brown hyena (**c**, **g**), and the striped hyena (**d**, **h**). bib – body of the incisive bone, iff – incisive foramina (green square marker), iic – interincisive canal, iis – interincisive suture, m – maxilla, nin – nasoincisive notch (black arrow in the spotted hyena and red square marker indicating absence of the nasoincisive notch in the brown hyena, striped hyena and aardwolf), npib – nasal process of the incisive bone, lsib – labial surface of the incisive bone, pf – palatal fissure (black arrow), ppib – palatine process of the incisive bone, psib – palatine surface of the incisive bone

**Fig. 14:**
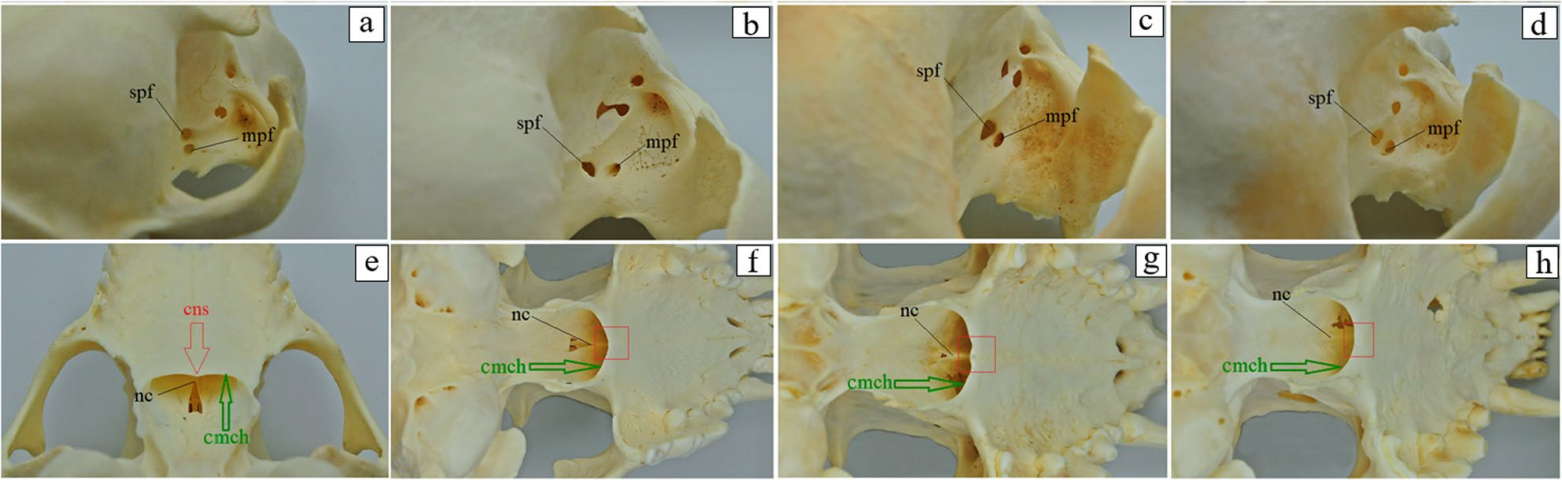
Palatine bone anatomy of the aardwolf (**a**, **e**) the spotted hyena (**b**, **f**), the brown hyena (**c**, **g**), and the striped hyena (**d**, **h**). cmch – caudal margin of the choanae (green arrow), cns – caudal nasal spine (red arrow – indicating the presence of the caudal nasal spine in the aardwolf and red square marker indicating the absence of the caudal nasal spine in the spotted hyena, brown hyena and striped hyena), mpf – major palatine foramen, nc – nasal crest, spf – sphenopalatine foramen

**Fig. 15:**
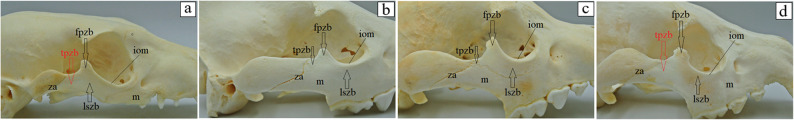
Zygomatic bone anatomy of the aardwolf (**a**) the spotted hyena (**b**), the brown hyena (**c**), and the striped hyena (**d**). fpzb – frontal process of the zygomatic bone (black arrow), iom – infraorbital margin, lszb – lateral surface of the zygomatic bone (black arrow), m – maxilla, tpzb – temporal process of the zygomatic bone (black arrow – indicates the minor contribution of this structure to the formation of the zygomatic arch in the spotted hyena and brown hyena, and red arrow indicates that the zygomatic arch is formed solely by the temporal process of the zygomatic bone, without the involvement of the maxilla), za – zygomatic arch

The external surface of the nasal bone is slightly concave in the aardwolf and the spotted hyena (Fig. 10e and f), whereas in the brown hyena and the striped hyena (Fig. 10g and h), it is more deeply concave. In all four species, the medial edge of the nasal bone curves into the nasal cavity, forming a distinct septal process (Fig. 10a-d). This process, together with its counterpart on the opposite side, provides attachment for the nasal septal cartilage. The rostral edge of the nasal bone is distinctly notched and rounded. It projects as a weakly developed nasal process in the aardwolf and the spotted hyena (Fig. 10a and b), while in the striped hyena and brown hyena (Fig. 12c and d), the nasal process is well developed. The lateral edge of this process borders a small nasoincisive notch in the spotted hyena (Fig. 10f). However, in the aardwolf, the brown hyena and the striped hyena, the nasoincisive notch is not visible (Fig. 10e, g and h). Only two surfaces of the lacrimal bone were distinguished in examined specimens: the orbital surface and the nasal surface (on the internal side) (Fig. 11a-d). The facial surface is absent. On the orbital surface of the lacrimal bone, near the orbital margin, a well-defined fossa is present for the lacrimal sac (Fig. 11a-d). At its base, a single round lacrimal foramen is observed, leading to the lacrimal canal (Fig. 11a-d). Caudal to the lacrimal sac fossa, on the orbital surface, there is an attachment site for the ventral oblique muscle (Fig. 11a-d). This attachment is represented by a single opening in the aardwolf, spotted hyena and the striped hyena (Fig. 13a, b and d), while in the brown hyena, two openings are visible (Fig. 13c). The maxilla forms the bony framework of the lateral facial wall. In the aardwolf and the brown hyena, the mean percentage involvement in the formation of the bony palate is as follows: 38.05% (23.82 mm) for the aardwolf and 42.87% (48.89 mm) for the brown hyena for the palatine process of the maxilla, 34.92% (21.86 mm) in the aardwolf and 30.95% (35.29 mm) in the brown hyena for the horizontal plate of the palatine bone, and 27.02% (16.92 mm) in the aardwolf and 26.17% (29.85 mm) in the brown hyena for the palatine process of the incisive bone (Fig. 11a and c). Meanwhile, the palatine process of the maxilla participates at a mean of 28.84% (33.75 mm) in the spotted hyena and in 18.85% (20.66 mm) in the striped hyena [value derived from a single specimen], it participates in the formation of the bony palate as the palatine process of the maxilla, together with the horizontal plate of the palatine bone – mean values of 37.12% (43.44 mm) and 37.79% (41.41 mm), and the palatine process of the incisive bone (mean values of 34.04% [39.85 mm] and 37.12% [43.44 mm]) (Fig. 12b and d). In all our specimens, the maxilla consists of the body of the maxilla and the frontal process, which possesses the ethmoid crest for the attachment of the dorsal nasal concha (Fig. 12e-h). On the body of the maxilla, the following surfaces are distinguished: a small orbital surface, the largest facial surface, the pterygopalatine surface, and the nasal surface (Fig. 12e-h). The facial surface is characterized by the absence of a facial crest, and the infraorbital foramen is located above the third cheek tooth, which, through a short infraorbital canal (5.391 ± 0.04 mm in the aardwolf ,14.75 ± 0.05 mm in the spotted hyena, 11.491 ± 0.1 mm in the brown hyena and 11.565 ± 0.2 mm in the striped hyena), opens through the maxillary foramen on the pterygopalatine surface (Fig. 12i-l). The body of maxilla in the spotted hyena and brown hyena participates in the formation of the zygomatic arch, whereas this association is not observed in the aardwolf and the spotted hyena (Fig. 12e-h). In the aardwolf, the brown hyena and the striped hyena, the caudal palatine foramen is located on the perpendicular plate of the palatine bone (Fig. 12i, k and l). However, in the spotted hyena, it is present directly on the palatomaxillary suture, leading to the major palatine canal and opening on the palatine process of the maxilla as the major palatine foramen (Fig. 12j). The major palatine foramen is located at the level of the first premolar tooth in the aardwolf, while in the spotted hyena, the brown hyena and the striped hyena, it is placed at the level of the second premolar tooth (Fig. 12m-p). Additionally, on the palatine process of the maxilla, 10 to 14 minor palatine foramina are observed in the aardwolf, 8 to 9 in the spotted hyena, 16 to 22 in the brown hyena, and 17 to 18 in the striped hyena (Fig. 12m-p). A distinct major palatine sulcus is also observed (Fig. 12m-p). The left and right palatine processes of the maxilla are joined by a prominent median palatine suture, which, extending rostrally, does not directly form the palatine fissure (Fig. 12m-p). Within the interincisive suture, 1 foramen is visible in the aardwolf, 10 foramina in the spotted hyena, 16 foramina in the striped hyena and 10 foramina in the brown hyena, leading to the interincisivus canal (Fig. 13a-h). The nasal process of the incisive bone, which is in contact with the maxilla, reaches the nasal bone, where it forms a small nasoincisive notch in the spotted hyena (Fig. 13b). However, in the aardwolf, the brown hyena and the striped hyena, this notch is not visible (Fig. 13a, c and d). The palatine process of the incisive bone has two distinct palatal fissures arranged obliquely in all hyenas studied. This is most prominent in the spotted hyena (Fig. 13f), to a lesser extent in the other three species (Fig. 13e, g and h). The palatine bone, in the aardwolf, the brown hyena and the striped hyena, has a large sphenopalatine foramen, located directly above the major palatine foramen (Fig. 14a, c and d). However, in the spotted hyena, the sphenopalatine foramen is located more caudally than the major palatine foramen (Fig. 14b). In the aardwolf, the nasal crest forms a caudal nasal spine, which is weakly marked (Fig. 14e), while in the spotted hyena, brown hyena, and striped hyena, the nasal spine is not present (Fig. 14f-h). The caudal margin of the choanae is rounded in the aardwolf, the brown hyena and the striped hyena (Fig. 14e, g and h), while in the spotted hyena it is sharp (Fig. 14f). In the examined specimens, the zygomatic bone connects to the maxilla via the temporal process of the zygomatic bone (Fig. 15a-d). It has a large lateral surface, which forms a small frontal process of the zygomatic bone, creating an open-type orbit (Fig. 15a-d). The infraorbital margin is sharp and narrow in the aardwolf (Fig. 15a), while the orbital and lateral surface in the spotted, brown, and striped hyenas form a wide and blunt infraorbital margin (Fig. 15b-d).

### Morphological differences and similarities of the mandible

**Fig. 16:**
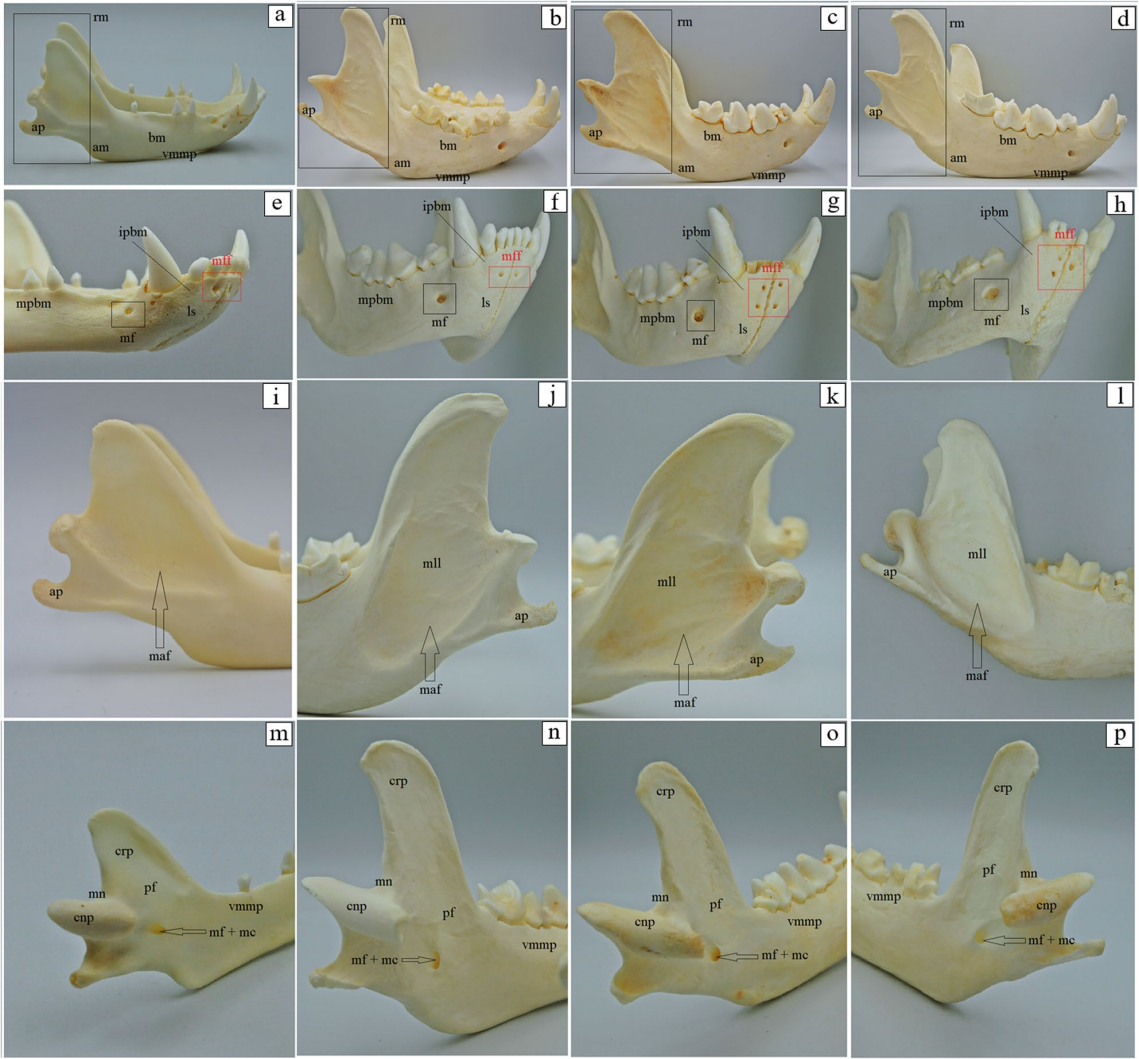
Mandibula anatomy of the aardwolf (**a**, **e**,** i**, **m**) the spotted hyena (**b**, **f**, **j**, **n**), the brown hyena (**c**, **g**, **k**, **o**), and the striped hyena (**d**, **h**, **l**, **p**). am – angle of the mandible, ap – angular process, bm – body of the mandible, cnp – condyloid process, crp – coronoid process, ipbm – incisive part of the body of mandible, ls – labial surface, maf – masseteric fossa (black arrow), mf + mc – mandibular foramen and mandibular canal, mf – mental foramen (black square marker), mff – mental foramina (red square marker), mll – muscular lines, mn – mandibular notch, mpbm – molar part of the body of mandible, pf – pterygoid fossa, rm – ramus of the mandible (black square marker), vmmp – ventral margin of the molar part

One pair of mental foramina is observed in the aardwolf and the spotted hyena (Fig. 16e and f). In contrast, in the brown hyena, two pairs of foramina are present (Fig. 16g), while in the striped hyena, three foramina are observed (two on the right side and one on the left side of the synchondrosis) (Fig. 16h). On the molar part of the body of the mandible, a variable number of mental foramina is observed on both sides: in the aardwolf – three foramina on the right side and two foramina on the left side (Fig. 16e); in the striped hyena – one foramen on both sides (Fig. 16h); in the brown hyena – one foramen on both sides (Fig. 16g). In four of five spotted hyenas – two foramina on the right side and one foramen on the left side (Fig. 16f), while in the fifth specimen (H/CC/1/C) – one foramen on the right side and two foramina on the left side. The ventral margin of the molar part, when observed from the side, is straight in the aardwolf, the spotted hyenas, the striped hyena (Fig. 16a, b and d), whereas in the brown hyena it is slightly convex (Fig. 16c). In the aardwolf no muscular lines on the ramus of the mandible are present. However, in the spotted hyena, brown hyena, and striped hyena, there is a deep masseteric fossa on the ramus of the mandible (Fig. 16i-l), with distinct longitudinal muscular lines (5–7 in the spotted hyena, 6–8 in the brown hyena, 6–7 in the striped hyena) and transverse muscular lines (2–3 in the spotted hyena, 1–2 in the striped hyena, and 6–11 in the brown hyena). The angle of the mandible has an angular process, which is shorter in the aardwolf and the brown hyena (Fig. 16m and o), when compared to the spotted hyena and the striped hyena (Fig. 16n and p).

### Morphometric observations

The analysis of 64 parameters revealed distinct differences in size, proportions and variability (Table 4). The analysis of the skull indices is presented below.

- I1: Skull index = Maximum zygomatic width (P32) x 100 / Skull length (Akrokranion - Prosthion) (P1).

The aardwolf exhibits a significantly lower skull index (56.01) compared to the other three hyena species. This indicates that the aardwolf has a relatively narrower skull relative to its length. The means of the skull indices for the spotted hyena (67.03), brown hyena (66.81), and striped hyena (65.73) are quite similar. This suggests that the proportions of the skull (width relative to length) are broadly similar. The aardwolf stands out with a proportionally narrower skull.

- I2: Facial index 1 = Maximum zygomatic width (P32) x 100 / Viscerocranial length (Nasion-Prosthion) (P7).

The aardwolf has a considerably lower facial index (133.16) compared to the bigger hyenas. This indicates that, relative to the length of its viscerocranium, the aardwolf has a narrower skull. The facial indices for the brown, striped and spotted hyenas are considerably higher (180.20, 177.44, and 166.74, respectively), suggesting relatively wider skulls compared to their facial length. The aardwolf shows a markedly different facial proportion, being relatively narrower for its facial length.

- I3: Facial index 2 = Greatest palatal breadth (P36) x 100 / Greatest length of the nasals (P9).

The spotted hyena exhibits the highest mean value (185.56), indicating it has a proportionally wider skull relative to its maxillary length compared to the other species. The striped hyena holds an intermediate position with a mean of 155.65, while the brown hyena has a proportionally narrower skull at 128.41. The aardwolf has the lowest mean index (85.42), signifying its skull is the most proportionally narrow in relation to maxillary length.

- I4: Index 1 = Maximum neurocranium width of neurocranium (P31) x 100 / Skull length (Akrokranion - Prosthion) (P1).

The aardwolf has the highest mean index (34.47), indicating that it has a relatively broad neurocranium (braincase) compared to its overall skull length. The spotted hyena has the next highest mean index (29.97), suggesting a moderately broad neurocranium in relation to its skull length. The next is the striped hyena (27.49), whereas the brown hyena has the lowest mean index (23.65), indicating a relatively narrower neurocranium compared to its overall skull length.

- I5: Index 2 = Maximum width of neurocranium (P31) x 100 / Basal length (Prosthion - Basion) (P3).

The aardwolf has the highest mean index (36.68), indicating that it has a relatively broad neurocranium compared to its basal length. Similarly to Index 1, the aardwolf has a proportionally broader braincase relative to its basal length. The brown hyena has the proportionally narrowest braincase (28.38). The spotted (34.34) and striped (31.83) hyenas fall in between.

- I6: Index 3 = Maximum width of neurocranium (P31) x 100 / Condylobasal length (Prosthion-Caudal border of the occipital condyles) (P2).

The aardwolf again has the highest mean index (34.92), indicating a relatively broad neurocranium compared to its condylobasal length. While the brown hyena has the lowest mean index (26.75), suggesting a relatively narrower neurocranium compared to its condylobasal length. The pattern continues: aardwolf with the relatively broad braincase, followed by spotted hyena (31.88), striped hyena (30.21), and then brown hyena with the relatively narrowest. Interspecific trends remain consistent across all three indices using different measures of skull length (total, basal, and condylobasal). The aardwolf consistently shows a relatively broader neurocranium, and the brown hyena a relatively narrower one. The specific index values fall between those of Index 1 (using total length) and Index 2 (using basal length), which is expected, as condylobasal length is generally intermediate in value between these two. The consistent pattern across different skull length measurements strengthens the conclusion that the aardwolf has a proportionally broader braincase relative to its overall skull dimensions compared to the other hyenas. The brown hyena consistently shows a relatively narrower braincase by these measures.

- I7: Foramen magnum index = Height of the foramen magnum (P30) x 100 / Maximum width of the foramen magnum (P29).

The aardwolf has the lowest mean index (69.60), indicating a relatively wider and less tall (more rounded) foramen magnum compared to the other species. The striped hyena, based on a single specimen, has the highest foramen magnum index (105.77). This suggests a relatively tall and narrow (oval) foramen magnum in this individual. The brown hyena has the next highest mean index (94.68), indicating a more oval shape compared to the spotted hyena and the aardwolf. The spotted hyena has a mean index of 88.14, suggesting a somewhat less elongated foramen magnum than the striped and brown hyenas.

### Multivariate morphometric analysis

**Fig. 17:**
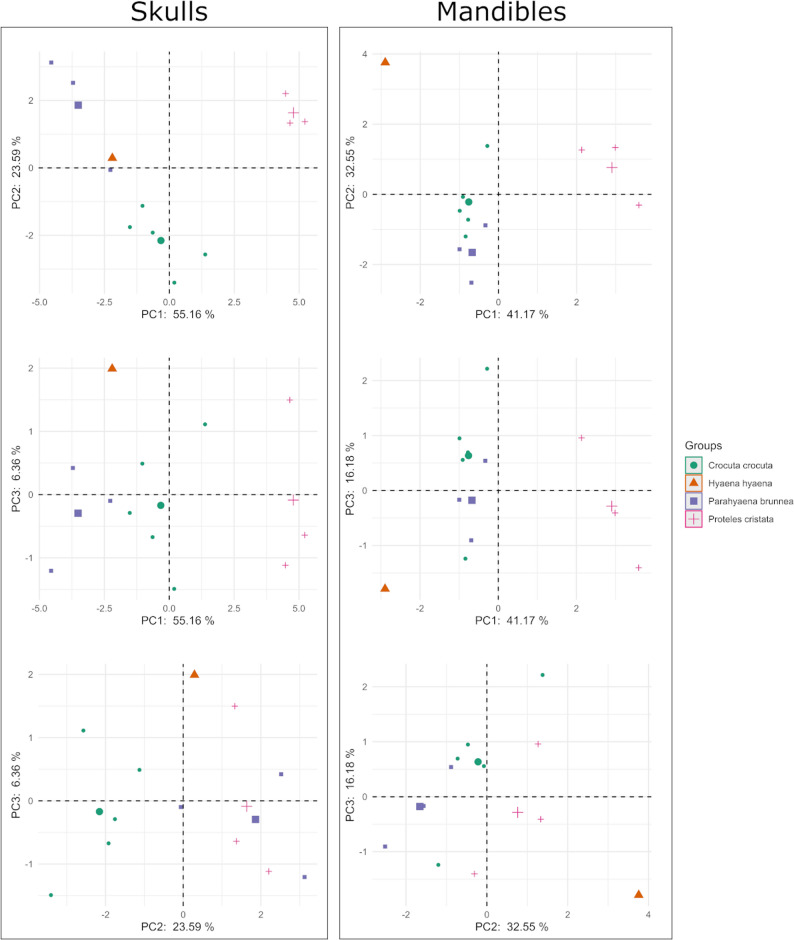
PCA scatter plot of the four hyenas: the aardwolf (*Proteles cristata*), the spotted hyena (*Crocuta crocuta*), the brown hyena (*Parahyaena brunnea*) and the striped hyena (*Hyaena hyaena*), based on skull (left) and mandible (right) measurements scaled to total cranial length (P1) and total mandibular length (P45), respectively

For the scaled cranial dataset (Table 5), the first two principal components account for 78.75% of the total variance (PC1: 55.16%, PC2: 23.59%). PC1 is strongly driven by parameters associated with cranial robustness and width, such as the zygomatic width (P10, loading 0.93) and the width of the postorbital constriction (P6, loading − 0.75). This axis clearly separates the robust, bone-crushing spotted hyena from the more slender-skulled aardwolf (Fig. 17, top left panel). PC2 captures variation in the facial and palatal regions. High loadings for the length of the viscerocranium (P34, loading 0.75) and neurocranium (P33, loading 0.94) allow for a distinct separation between striped and brown hyenas. The inclusion of PC3 (Fig. 17, middle and bottom left panels) further refines the clustering, highlighting subtle differences in the palatal width (P30, loading 0.74), which differentiates the specialized insectivorous aardwolf from the opportunistic brown hyena.

**Table 5:** Results of the PCA on scaled skull measurements, displaying the eigenvalues and cumulative variance percentage for the first five principal components (PC1-PC5), and the factor loadings for the analyzed parameters

	PC1	PC2	PC3	PC4	PC5
eigenvalues	9.93	4.25	1.14	0.85	0.64
cumulative variance percentage	55.16	78.75	85.11	89.86	93.41
loadings:					
P2	0.94	-0.2	0.01	0.02	0.19
P3	0.96	0.04	0.07	-0.01	0.2
P6	-0.75	-0.58	0.08	0.24	0.03
P8	0.92	-0.06	-0.18	-0.07	-0.12
P10	0.93	-0.13	0.14	0.14	-0.01
P27	0.69	-0.3	-0.37	0.52	0.02
P29	0.94	0.01	0.06	0.12	-0.14
P30	0.29	-0.38	0.74	0.27	-0.25
P31	0.93	-0.26	0.02	0.12	0.18
P33	0.94	0.23	0.03	0.17	-0.02
P34	0.75	0.47	0.21	-0.34	0.03
P35	-0.05	-0.81	-0.35	-0.16	-0.1
P36	-0.11	-0.94	-0.04	0.02	0.3
P39	0.74	-0.51	-0.1	-0.27	0.18
P40	0.91	-0.1	0.17	-0.25	0.06
P41	0.6	-0.46	-0.28	-0.1	-0.52
P42	-0.59	-0.73	0.21	0	0.11
P43	-0.07	-0.84	0.25	-0.28	-0.14

*P* Parameter, *PC* Principal component

In the mandibular analysis (Table 6), the first three components explain nearly 90% of the variance (PC1: 41.17%, PC2: 32.55%, PC3: 16.18%). PC1 is primarily influenced by the height of the mandible (P59, P60, P61; all loadings < -0.85). This axis reflects the biomechanical strength of the lower jaw, separating the hypercarnivorous spotted hyena from the aardwolf. PC2 is dominated by the length of the dental row and the position of the mental foramen (P44, P47; loadings − 0.83). This component successfully distinguishes the brown hyena from the striped hyena, reflecting differences in dental crowding and jaw elongation. The separation in the mandibular morphospace (Fig. 17, right panels) clearly aligns with the dietary specializations of the four species, ranging from the delicate jaw of the termite-eating aardwolf to the massive, crushing apparatus of the spotted hyena.

**Table 6:** Results of the PCA on scaled mandible measurements, displaying the eigenvalues and cumulative variance percentage for the first five principal components (PC1-PC5), and the factor loadings for the analyzed parameters

	PC1	PC2	PC3	PC4	PC5
eigenvalues	3.29	2.6	1.29	0.48	0.23
cumulative variance percentage	41.17	73.72	89.91	95.85	98.71
loadings:					
P44	-0.05	-0.83	-0.39	0.35	-0.15
P46	0.54	-0.73	-0.16	-0.35	0.12
P47	-0.29	-0.83	-0.41	-0.05	0.08
P48	-0.48	0.48	-0.65	-0.32	0.04
P49	-0.66	0.48	-0.52	0.23	0.05
P59	-0.87	-0.16	0.18	-0.27	-0.32
P60	-0.85	-0.41	0.31	0.01	0.03
P61	-0.87	-0.19	0.34	0.05	0.28

*P* Parameter, *PC* Principal component

**Fig. 18:**
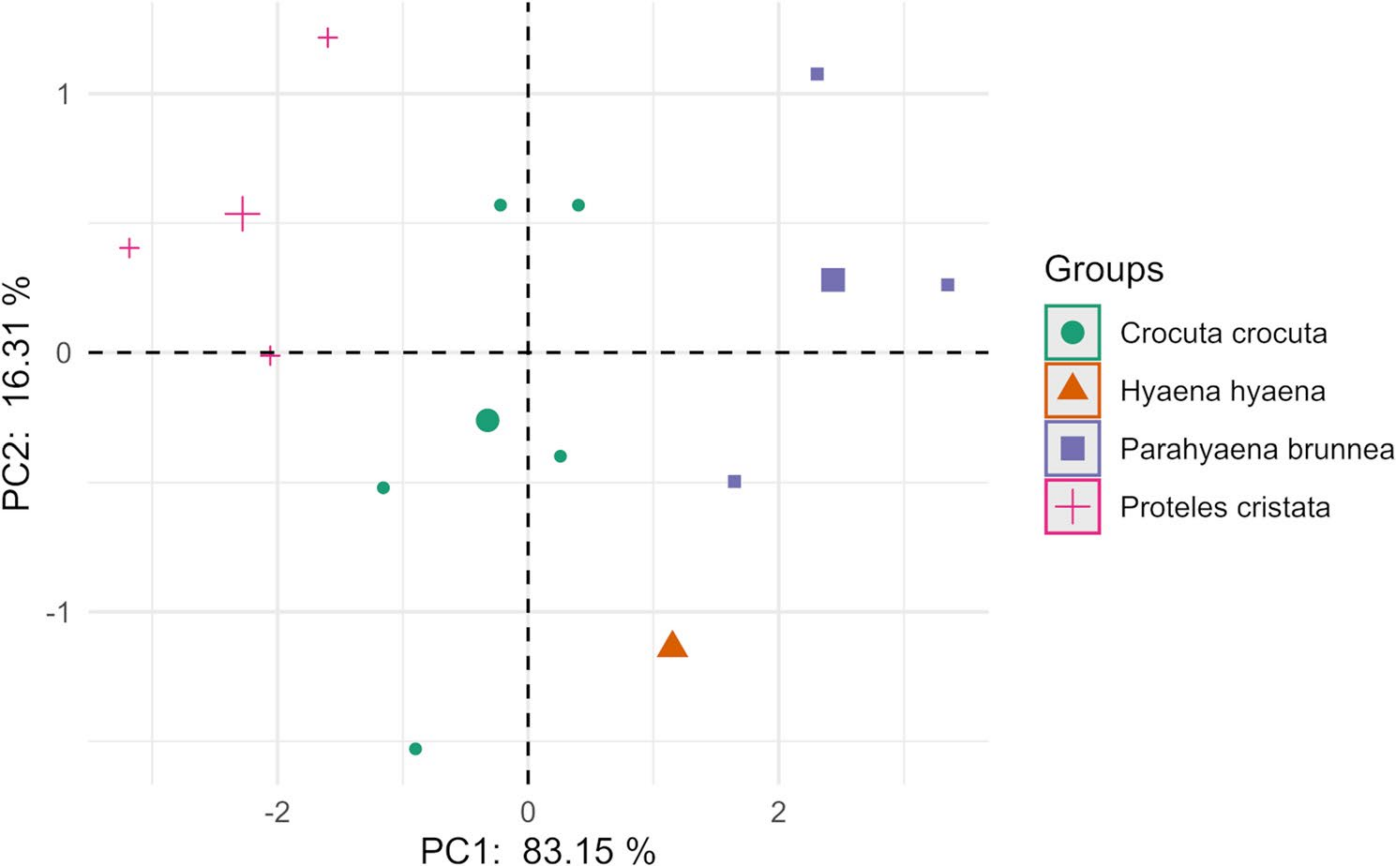
PCA scatter plot of the four hyenas: the aardwolf (*Proteles cristata*), the spotted hyena (*Crocuta crocuta*), the brown hyena (*Parahyaena brunnea*) and the striped hyena (*Hyaena hyaena*), based on craniometric shape indices

The PCA on craniometric indices (Fig. 18; Table 7) remains a powerful indicator of specialization. PC1 (98.39%) continues to isolate the aardwolf as a significant outlier within the hyaenids. This extreme separation is driven by indices reflecting the drastic reduction of the masticatory apparatus. PC2 (0.7%), while capturing a smaller fraction of variance, provides a secondary axis that differentiates the spotted hyena’s bone-crushing adaptations from the more generalized proportions of the striped and brown hyenas.

**Table 7:** Results of the PCA on calculated shape indices, displaying the eigenvalues and cumulative variance percentage for the first four principal components (PC1-PC4), and the factor loadings for the analyzed indices

	PC1	PC2	PC3	PC4
eigenvalues	8.85	0.06	0.05	0.03
cumulative variance percentage	98.39	99.09	99.62	99.91
loadings:				
I4	-0.99	-0.13	-0.07	0.05
I5	-0.97	-0.22	0.09	0.01
I6	-0.98	-0.16	-0.03	-0.06
I7	0.66	-0.75	-0.01	0

*I* Index, *PC P*rincipal component

## Discussion

Our study offers a detailed analysis of the aardwolf’s skull anatomy and craniometry, with a comparison to the spotted, brown, and striped hyenas using specimens from Polish and South African collections. We revealed numerous morphological and morphometric differences between the aardwolf’s skull and the other three species.

The occipital bone, which forms the caudal base of the cranium, serves as a critical site for muscle attachments and neurological connections. We observed variations in the shape of the basilar part. Although the functional implications of the shape of the basilar part (cylindrical vs. pyramidal) require further investigation, it should be noted that the overall robustness of the occipital region is a common feature in Carnivora that engage in powerful biting or specialized feeding behaviors [13, 45–49]. For example, many ursids, known for their strong bite force, also exhibit robust occipital regions [17, 46–53]. This contrasts with some felids, where the occipital region, while still important for muscle attachment, may be relatively less massive compared to their overall skull structure, reflecting their primary reliance on precise, killing bites rather than bone crushing [17, 45–49, 52]. The presence of a jugular foramen rather than a foramen lacerum in the hyenas is a specific anatomical detail. The functional and evolutionary significance of this variation within Carnivora warrant further exploration, potentially involving comparative studies across a wider range of species. Muscle tubercles on the basilar part, serving as attachment sites for neck muscles, are particularly well defined in the spotted hyena. This observation aligns with the powerful neck musculature of the spotted hyena, crucial for prey manipulation and bone crushing [15, 28, 30]. On the contrary, the reduced muscular tubercles in the brown and striped hyenas and their absence in the aardwolf likely reflect differences in the feeding ecology. Aardwolves, which primarily feed on termites, do not require the same degree of neck muscle strength as their bone-crushing relatives. The morphology of the foramen magnum, the opening for the spinal cord, is remarkably consistent between the studied species, yet varies in size. This variation in size is generally correlated with overall skull size and, by extension, brain size [54]. However, subtle variations in the shape of the foramen magnum could potentially influence head movement and posture, areas that could benefit from further comparative study within Carnivora.

The temporal bone, which contributes to the zygomatic arch, is essential in carnivore feeding. The zygomatic arch, formed by the temporal and zygomatic bones, is a key determinant of the force of bite. A more robust zygomatic arch allows for the attachment of larger masseter muscles, generating greater force [15, 28, 30]. Hyenas, especially spotted and brown hyenas, are known for their powerful bites, enabling them to crush bones and consume a wide range of food items [9]. This is reflected in their strong zygomatic arches. Felids, while also possessing strong bites, often have a different emphasis in their masticatory apparatus. Their skulls and jaw musculature are adapted for delivering a precise and powerful killing bite, often targeting the neck of their prey [46, 55–59]. Canids, with their more varied diets, exhibit a range of zygomatic arch development. Wolves, for instance, have strong jaws for hunting and consuming larger prey, while smaller canids like foxes have more gracile skulls [16, 30, 46, 60, 61].

Parietal bones and frontal bones, which form the cranial vault and parts of the face, also reveal important adaptations. The sagittal crest, a prominent ridge in the parietal bones, serves as a crucial attachment site for the temporal muscle. Its size is directly related to the size of the muscle and, therefore, to the bite force. The well-developed sagittal crest in spotted, brown, and striped hyenas is a clear indicator of their powerful bite and bone crushing abilities. On the contrary, the reduced sagittal crest of the aardwolf is consistent with its weak bite, sufficient to consume termites [8, 9, 11, 12]. The frontal bones contribute to the structure of the orbits and the rostral part of the skull. Variations in features such as the supraorbital margin and the presence or absence of a supraorbital foramen can reflect differences in facial structure and soft tissue arrangement. The position and size of the orbits can also provide clues about the visual capabilities and hunting strategies of the animal. For example, felids, with their reliance on vision for hunting, tend to have relatively large orbits [46, 59, 62].

The nasal region and the palate are also subject to evolutionary pressures related to diet and sensory function. The length and shape of the nasal bones can influence the size and shape of the nasal cavity, which plays a role in olfaction [63]. Canids, with their strong dependence on smell, typically have elongated nasal regions [64, 65]. Hyenas, while possessing a good sense of smell, do not rely on it to the same extent as canids and exhibit variations in nasal bone morphology [66, 67]. The palate, which forms the roof of the mouth, is crucial for food processing. Its width, length, and presence of features such as palatine ridges can vary depending on the animal’s diet. Hyenas, with their varied feeding habits, show corresponding variations in palatal morphology.

The mandible is the main driver of the bite force and chewing mechanics. We provide a detailed description of its various parts and features. The height and robustness of the mandible, as well as the size and shape of the muscle attachment processes, are directly related to the force of the bite. Hyenas, particularly spotted and brown hyenas, possess deep and robust mandibles, allowing the attachment of powerful jaw muscles. This morphology enables them to generate the force necessary to crush bones and consume large amounts of food [15, 28, 30]. The aardwolf, again, stands out with its gracile mandible, reflecting its specialized diet and reduced bite force [8, 9, 11, 12].

## Conclusions

The morphometric analysis presented in this study provides valuable quantitative data to support the observed morphological diversity within the Hyaenidae. The results of the PCA clearly demonstrated that the four extant hyena species occupy distinct and non-overlapping regions of the morphospace, confirming a fundamental cranial and mandibular divergence driven by dietary specializations. The most striking adaptations were observed in the aardwolf, which remains a significant outlier in all multivariate analyses. Its delicate skull and strongly reduced cheektooth row reflect a drastic departure from the ancestral hyaenid condition in response to a specialized myrmecophagous diet. Its cranial and mandibular morphology is fundamentally divergent from that of the bone-crushing species. In contrast, the powerful bone-crushing apparatus of spotted and brown hyenas, characterized by high cranial robustness and increased mandibular height, directly correlates with their roles as apex predators and efficient scavengers capable of durophagy. The smaller skull of the striped hyena and its intermediate morphological features occupy a unique position in the morphospace that reflects its more varied, opportunistic diet.

Future research employing increased sample sizes in morphometric analyzes, as well as the incorporation of 3D modeling, biomechanical simulations and ontogenetic investigations of skull development, would contribute to a more precise analysis of the functional morphology of cranial and mandibular structures. Integrating these methodological approaches with genetic data has the potential to yield a more complete depiction of the evolutionary trajectory of hyenas and their ecological significance within the order Carnivora.

## Data Availability

The data and materials are available from the corresponding authors upon reasonable request.
